# Adsorptive performance of SPE via modified POM biochar for Pb(II) and tetracycline with concurrent antimicrobial action

**DOI:** 10.1038/s41598-025-33048-w

**Published:** 2026-01-12

**Authors:** Mohamed S. Abdelwahab, Mohamed E. Mahmoud, Rasha A. Metwally

**Affiliations:** 1 Faculty of EducationPhysics and Chemistry Department, Matrouh University, Mersa Matrouh, Egypt; 2 Faculty of Science, Chemistry Department, Alexandria University, Alexandria, Egypt; 3 Marine Microbiology Lab, National Institute of Oceanography and Fisheries, NIOF, Alexandria, Egypt

**Keywords:** Solid phase extraction (SPE), Tetracycline, Pomegranate, Biochar, Antimicrobial, Water treatment, Lead, Biotechnology, Chemistry, Environmental sciences

## Abstract

This study developed a novel, eco-friendly solid-phase extraction (SPE) sorbent, modified pomegranate biochar (POM), from the agricultural waste of pomegranate peel for the efficient removal of toxic lead (Pb(II)) and the tetracycline (TC) antibiotic from aqueous solutions. Synthesized POM was comprehensively characterized using (SEM, TEM, FT-IR, and XRD) and confirmed POM’s hierarchical core-shell nanostructure, enriched surface functional groups. Optimized SPE conditions using POM achieved maximum adsorption capacities of 3900 ± 71 (RSD = 1.8%, *n* = 3) µmol/g for Pb(II) at (pH 7, 20 s microwave-assisted) and 87.6% ± 0.18 (RSD = 0.001%, *n* = 3) removal for TC at (pH 4, 30 min shaking). while PO achieved (2000 ± 71 (RSD = 2.8%, *n* = 3) µmol/g Pb(II); and 45.9% ± 1.8 (RSD = 0.03%, *n* = 3) TC removal). Although the pseudo-second-order (PSO) model accurately describes the kinetic data for Pb(II) adsorption on POM and PO, this alone does not prove a chemisorption mechanism. In general, the thermodynamic parameters, especially ΔH°, have proved that the primary mechanism is physisorption; nevertheless, the excellent fit to the PSO model suggests that the adsorption process may incorporate certain characteristics of chemisorption or that the rate-limiting step involves the sharing or exchange of electrons. While TC obeys the pseudo-first order model, indicating a physisorption mechanism for both POM and PO. Additionally, the Langmuir model provides the best fit for both POM and PO with Pb(II) and TC, indicating the formation of a monolayer on the adsorbent surface. Efficient desorption used 1 M HCl for Pb(II) (98.7% recovery) and acidified methanol (1:1 v/v HCl: methanol) for TC (99.3% recovery). The Limit of Detection (LOD) and limit of quantification (LOQ) for TC were 1.725 ppm and 5.227 ppm, while Pb(II) ions were 16.27 ppm and 50.06 ppm, respectively. The thermodynamic parameters for the adsorption of Pb(II) and TC onto both POM and PO confirm a spontaneous and endothermic process driven by an increase in entropy. POM also exhibited antibacterial activity comparable to that of a standard antibiotic, particularly against Gram-negative bacteria (*E. coli* and *P. aeruginosa*).

## Introduction

 Solid-phase extraction (SPE) is a separation and concentration technique that involves the adsorption of target analytes onto the surfaces of various sorbents from liquid or gas phases, followed by elution or thermal desorption. The SPE approach is highly sought after due to its modifiability, such as the incorporation of coordination polymers (CPs) that can interact with antibiotics and produce supramolecular complexes, which are not well understood and can exhibit luminescence under specific conditions^1^. In addition, the SPE method can be easily automated to conduct real-time analysis by integrating it with other analytical techniques, such as gas or liquid chromatography, photoluminescence, and others. The automation possibilities and compatibility of SPE with gas and liquid chromatography, as well as other analytical techniques, have led to an increasing demand for it^2^. A cost-efficient alternative to costly SPE cartridges is the utilization of packed SPE cartridges. This method involves carefully loading a cartridge with a predetermined amount of sorbent, then passing a liquid sample through it to facilitate the adsorption and retention of the desired analytes. The next step is to use the available solvents to conduct the elution^3^. Dispersive solid-phase extraction involves incorporating a sorbent into the sample, vigorously agitating the mixture, separating the sorbent via centrifugation, and subsequently eluting it.

Lead (Pb) is a hazardous heavy metal when present in water. The term “heavy metals” refers to metals that are toxic to living organisms, even in small amounts, due to their high density (typically greater than 5 g/cm³). Lead is a particularly ubiquitous and toxic contaminant, infamous for its capacity to bioaccumulate in living organisms. It is dangerous because it can mimic key metals, which means it can interfere with essential enzyme functions, halt neurodevelopment, and harm vital organs such as the brain and kidneys. Infants are especially vulnerable since exposure can cause long-term brain damage^4^. In recognition of these significant health hazards, global health organizations have implemented stringent regulations on lead levels in drinking water. The World Health Organization (WHO) stipulates a maximum allowable concentration of 0.01 mg/L (10 µg/L) in its Guidelines for Drinking-water Quality. The advancement of effective and cost-efficient remediation methods is not merely a scientific endeavor, but a public health necessity to ensure water safety and comply with essential regulatory criteria, which serves as the primary impetus for this investigation into Pb(II) removal^5^. Lead is poisonous to humans if present at any level^6^. Also, there is no actual safe level of blood lead, especially in children. Lead affects the growth, hearing, blood, and IQ of children. Lead, similar to calcium, builds up in our systems as we age^6^. During pregnancy, lead is liberated from the mother’s bones and ends up in the fetus’s bones. Additionally, lead can accumulate in crops and grains if they are irrigated with contaminated irrigation water containing lead^7^.

The tetracycline (TC) family of broad-spectrum antibiotics is essential in aquaculture, veterinary medicine, and human health because it kills a wide range of bacteria. Up to 90% of doses are excreted unmetabolized through urine and feces due to their extensive use and improper management, resulting in their widespread release into water systems. Inadequately treated municipal sewage, agricultural runoff from fertilized fields, pharmaceutical manufacturing waste streams, aquaculture effluent discharge, industry, and other sources allow these residues to enter aquatic ecosystems^8,9^. The intrinsic resistance to biodegradation and the stable tetracyclic naphthalene core of tetracyclines ensure that they persist once injected into water bodies. Contamination of surface water, groundwater, and even drinking water sources occurs as a result of this environmental persistence, with observed amounts ranging from nanograms to micrograms per liter. The ongoing presence of TC has a selective pressure that encourages the growth of ARBs (Antibiotic-Resistant Bacteria) and disturbs vital microbial communities that support the health of aquatic ecosystems. To mitigate these significant threats to public health and the environment, it is crucial to thoroughly remove tetracycline from water and wastewater. An assortment of sorbents has been developed to extract these dangerous compounds from water^9^.

Preparing an eco-friendly sorbent is mandatory to prevent any side effects of the chemicals used in the synthesis methods. A plentiful agricultural waste, pomegranate peel, is transformed into a high-performance biochar sorbent (PO), which utilizes the notion of a circular economy. By redirecting biomass away from landfills, decreasing reliance on synthetic adsorbents, and establishing a closed-loop system from waste to water treatment, this method offers substantial sustainability benefits while transforming a low-value byproduct into a helpful resource for environmental rehabilitation. The lifecycle of PO conserves natural resources, lowers the carbon footprint of remediation technologies, and adds economic value to agricultural waste, aligning with green chemistry principles^10^.

Pomegranate (*Punica granatum L*.) trees were first cultivated by humans; their use has been limited due to the difficulty of removing the seeds^11–13^. The advancement of scientific studies highlighting the benefits of pomegranates has led to a significant increase in pomegranate production and consumption^14–17^. In addition to being consumed fresh, pomegranate fruits are processed into numerous products, including juice, oil, wine, and preserves^11,14,18^. There are many different kinds of phytochemicals found in fruits and fruit peels. Some examples are tannins, phenolic acids, and flavonoids. It is thought that phytochemicals have many components that increase their powerful antioxidant capabilities and health benefits^14,19–21^. A large amount of waste products is generated during the peel extraction process, which is characterized by its high levels of hydrolyzable tannins^12,18,22^. Pomegranate peel extract (PPE) has gained particular popularity in recent years due to its purported medicinal benefits. These characteristics include the ability to reduce inflammation, ulcers, and bacterial growth, as well as fight cancer and other diseases^19,21–24^. Numerous studies indicate that PPE can significantly reduce the risk of food poisoning and extend the shelf life of food after harvest. Pomegranate peel waste can be converted into biochar, which serves several environmental purposes^25^.

Biochar is produced through the pyrolysis of agricultural and forestry residues in an environment with minimal or no oxygen^26–29^. This effort aims to generate pomegranate biochar for the sorption of heavy metals^30–35^. In recent years, biochar has gained popularity as a heavy metal adsorbent due to its large surface area, high porosity, and pH, as well as the presence of active functional groups, including hydroxyl, carboxyl, and carbonyl groups^36^. To a large extent, biochar’s physical properties determine its adsorption values. The feedstock and pyrolysis factors, such as temperature, have the most significant impact on the characteristics of the biochar. A variety of materials were examined as prospective feedstocks for biochar production, including agricultural residues, animal waste, industrial byproducts, and sewage sludge, among others^34^.

In this research, we present a novel sorbent with triple-action remediation potential that is both ecologically friendly and versatile, made from discarded pomegranate peel. In addition to the material’s inherent broad-spectrum antibacterial activity, the breakthrough lies in the feasible, contemporaneous SPE of TC and lead(II). This comprehensive approach tackles the major problem of co-contaminated wastewater head-on, where conventional sorbents could unintentionally promote the growth of bacteria resistant to antibiotics. This research is establishing a new paradigm for sustainable and comprehensive water treatment approaches, transforming agricultural waste into a high-performance polymer that addresses both chemical and biological impurities. Examining the adsorption of Pb(II) and TC across several factors, such as pH levels, contact durations, sorbent quantities, metal ion concentrations, eluent kinds, volumes, desorption timings, and acidification of eluents, is the main emphasis of this work. Scanning electron microscopy (SEM), transmission electron microscopy (TEM), Fourier transform infrared spectroscopy (FT-IR), and X-ray diffraction (XRD) were used to characterize the synthesized nanocomposite. The LOD and LOQ for the two pollutants were also determined.

## Experimental

### Materials

The ingredients used are pomegranate peel obtained from a local market, ethylenediaminetetraacetic disodium acetate dihydrate, hydrochloric acid, absolute ethyl alcohol, sodium acetate, acetone, lead nitrate, tetracycline, and DMSO, which were purchased from Alpha Chemicals, India, and American Type Culture Collection (ATCC) bacterial standard, reference strains of Gram-positive strains of *Staphylococcus aureus* (ATCC 25923) and *Bacillus subtilis* (ATCC 6633), as well as Gram-negative strains of *Escherichia coli* (ATCC 8739) and *Pseudomonas aeruginosa* (ATCC 9027), USA.

### Synthesis of modified pomegranate biochar (POM)

The modified pomegranate Biochar (POM) was prepared using the following procedures. PO is prepared using pomegranate peel and then muffled at 300 °C to produce the biochar. The pomegranate extract was prepared from 50 g of dried pomegranate peel powder and was immersed in 100 mL of pure acetone for 48 h under continuous stirring to extract phytochemicals from the pomegranate peel. POM was prepared using 25 g of PO, which was ground using a manual mortar with 100 mL of acetone extract to apply phytochemicals on the surface of the biochar. Subsequently, the sample was subjected to a drying process at 40 °C until it reached a state of total dryness.

### Characterization of POM

POM was analyzed using several techniques. Table 1 provides a comprehensive list of the instruments used, along with the specific criteria that must be met to identify them .

**Table 1:** Instrumental techniques and their specifications.

Instrument	Model	Operating conditions
Scanning electron microscope (SEM and EDX)	JSM-6360LA, JSM-5300JEOL, JEOL-JFC-1100E Ltd., USA	25KV, 25000X
Transmission electron microscope (TEM)	JEOL, JEM 1400, USA	80KX
XRD	X’Pert³ Powder, Netherland.	X-ray tube target: Cu voltage: 40.0 (kV), scan speed = 12.0000 (deg/min)
Microwave oven	Model KOG-1B5H, Korea	1400-W and 2.45 GHz.
FT-IR	BRUKER spectrophotometer	500–4000 cm^− 1^
GC- MS	Agilent 5977 C, USA	Scan mode

**Table 2:** Kinetic parameters of Pb(II) adsorption using POM and PO.

Metal ion	Pseudo-first-order parameters				
	**Sorbent**	**R** ^**2**^	**q**_**e**_ **(mmol/g)**	**q**_**exp**_	**k**_**1**_ **(min**^**− 1**^**)**
Pb(II)	POM	0.9722	4.1	1.4743	7.7907
	PO	0.9957	2.3	1.8775	5.0603
	**Pseudo-second-order parameters**				
	**Sorbent**	**R** ^**2**^	**q**_**e**_ **(mmol/g)**	**q**_**exp**_	**k**_**2**_ **(g/mg.min**^**− 1**^**)**
	POM	0.9944	4.1	4.3641	9.6145
	PO	0.9964	2.3	2.7879	2.7382
	**Elovich**				
	**Sorbent**	**R** ^**2**^	**β**	**α**	
	POM	0.8777	2.2907	204.721	
	PO	0.9916	1.6368	0.74495	
	**Intra particle diffusion**				
	**Sorbent**	**R** ^**2**^	**K**_**t**_	**C**	
	POM	0.8540	0.241761	2.84029	
	PO	0.9611	0.337676	0.341153	

### Solid phase extraction (SPE)

This study utilized various parameters to investigate the adsorption capacity of TC and lead ions via the SPE technique using POM and PO.

#### The pH effect

For Pb(II) adsorption, 10 mg of PO and POM were separately added to a measuring flask containing 9 mL of a buffer solution with a pH range of 1–7. One milliliter of a solution containing 0.1 M of Pb(II) was added to the mixture. The measuring flask was then exposed to radiation for 20 s. After that, the mixture was filtered and washed with DW. The concentration of the residual ions was measured by titration with a 0.01 M EDTA solution^37^.

For TC adsorption, 10 mg of PO and POM were added to a measuring flask containing 9 mL of buffer solution with a pH range of 1–7. A 1 mL solution containing 10 ppm TC was added to the mixture. The measuring flask was then exposed to shaking for 30 min at 200 rpm. After that, the mixture was filtered and washed with DW. The remaining TC concentration was determined using a UV-Vis spectrophotometer at a wavelength of 357 nm.

#### Time effect

Ten milligrams of PO and POM were individually put into a measuring flask. The volume of the optimum buffer required is 9 milliliters, where the optimum buffer has a pH of 7. One milliliter of 0.1 M Pb(II) metal ion was included. The measuring flask was exposed to microwave radiation for different durations (ranging from 3 to 40 s), followed by filtration. The remaining Pb(II) ions were titrated against a solution of EDTA (0.01 M).

For TC adsorption, 10 mg of PO and POM were added to a 9 mL measuring flask containing the optimum buffer solution. A 1 mL solution containing 10 ppm TC was added to the mixture. The measuring flask was then exposed to shaking (10, 20, 30, 40, and 50 min) at 200 rpm. After that, the mixture was filtered and washed with DW. The remaining TC concentration was determined using a UV-Vis spectrophotometer at a wavelength of 357 nm.

#### Dosage effect

For Pb(II) adsorption, the effects of metal dosage on sorption using the microwave technique have been investigated at various doses ranging from 5 to 100 milligrams under optimal buffer and time conditions. Next, the remaining Pb(II) was separated using filtration and purified using DW. The remaining Pb(II) was determined using 0.01 M EDTA.

Different doses (5, 10, 20, and 30 milligrams) of PO and POM were added to a measuring flask with an optimum buffer solution for TC adsorption. A 1 mL solution containing 10 ppm TC was added to the mixture. The measuring flask was then exposed to shaking for 30 min at 200 rpm. After that, the mixture was filtered and washed with DW. The remaining TC concentration was determined using a UV-Vis spectrophotometer at a wavelength of 357 nm.

#### Effect of pollutant concentration

For Pb(II) adsorption, 10 mg of PO and POM were placed in a measuring flask with 9 mL of optimal buffer and varying amounts of lead ions (ranging from 0.02 to 0.1 mol/L). The sorption procedure utilizing microwaves was concluded within 20 s, and the remaining Pb(II) was subjected to titration with a solution of EDTA (0.01 M).

For TC adsorption, 10 milligrams of PO and POM were added to a measuring flask containing the optimum buffer solution. One milliliter solution containing (5, 10, 20, 30, and 40 ppm) TC was added to the mixture. The measuring flask was then exposed to shaking for 30 min at 200 rpm. After that, the mixture was filtered and washed with DW. The remaining TC concentration was determined using a UV-Vis spectrophotometer at a wavelength of 357 nm.

#### Effect of eluent type

Using the optimum conditions for the adsorption process of Pb(II). The solid adsorbent was separated using a centrifuge at 5 min, 5500 rpm, then placed with 5 mL of different Eluents (methanol, ethanol, 1 M HCl, and 1 M HNO_3_) for 30 min, shaking at 200 rpm, followed by centrifuging for 5 min at 5500 rpm to study the desorption process and calculate the recovery percent.

Using the optimum conditions for the adsorption process of TC. The solid adsorbent was separated using a centrifuge at 5 min 5500 rpm, then placed with 5 mL of different eluents (methanol, ethanol, acetone, and hexane) for 30 min, shaking at 200 rpm, followed by centrifuging at 5 min 5500 rpm to study the desorption process and calculate the recovery percent.

#### Effect of eluent volume

Using the optimum conditions for the adsorption process of Pb(II). The solid adsorbent was separated using a centrifuge at 5 min 5500 rpm, then placed with different volumes of 1 M HCl (0.5, 1, 2, 3, 4, and 5 mL) for 30 min, shaking at 200 rpm, followed by centrifuging at 5 min 5500 rpm to study the desorption process and calculate the recovery percent.

Using the optimum conditions for the adsorption process of TC. The solid adsorbent was separated using a centrifuge at 5 min and 5500 rpm. It was then placed in 3, 4, 5, and 10 mL of different optimum eluents for 30 min of shaking at 200 rpm, followed by centrifugation at 5 min and 5500 rpm to study the desorption process and calculate the recovery percentage.

#### Effect of desorption time

Using the optimum conditions for the adsorption process of Pb(II). The solid adsorbent was separated using a centrifuge at 5 min 5500 rpm, then placed with 1 mL of 1 M HCl for different times (5, 10, 20, and 30 min), shaking at 200 rpm, followed by centrifuging at 5 min 5500 rpm to study the desorption process and calculate the recovery percent.

Using the optimum conditions for the adsorption process of TC. The solid adsorbent was separated using a centrifuge at 5 min 5500 rpm, then placed with 5 mL of optimum eluents for different times (5, 10, 20, and 30 min), shaking at 200 rpm, followed by centrifuging at 5 min 5500 rpm to study the desorption process and calculate the recovery percent.

#### Effect of HCl concentration

Using the optimum conditions for the adsorption process of Pb(II). The solid adsorbent was separated using a centrifuge at 5 min 5500 rpm, then placed with 1 mL of HCl with different concentrations (0.5, 1, 2, and 3 M), shaking at 200 rpm, followed by centrifuging at 5 min 5500 rpm to study the desorption process and calculate the recovery percent.

#### Effect of eluent acidification

Using the optimum conditions for the adsorption process of TC. The solid adsorbent was separated using a centrifuge for 5 min at 5500 rpm. Acidification of the optimum eluent with HCl significantly enhanced the desorption of TC. Different ratios of methanol: HCl mixtures (1:0.2, 1:0.6, and 1:1 v/v) were used at the optimum desorption time, with shaking at 200 rpm, followed by centrifugation for 5 min at 5500 rpm. This process was employed to study the desorption process and calculate the recovery percentage.

#### Effect of temperature

For Pb(II) adsorption, 10 mg of PO and POM were placed in a measuring flask containing 9 mL of the optimal buffer and 1 mL of a 0.1 M Pb(II) solution. This mixture was heated inside a microwave oven for 20 s, using power percentages corresponding to 10%, 30%, 50%, 70%, and 100%. The residual metal content in the filtrate was determined by titration against (0.01 M EDTA).

For TC adsorption, 10 mg of PO and POM were added to a measuring flask containing the optimum buffer solution. One milliliter of solution containing 10 ppm of TC was added to the mixture. The adsorption process was conducted at different temperatures (15, 20, 25, 30, 35, and 40 °C). The measuring flask was then exposed to shaking for 30 min at 200 rpm. Thereafter, the mixture underwent filtration and DW washing. The remaining TC concentration was determined using a UV-Vis spectrophotometer at a wavelength of 357 nm.

### Antibacterial activity of POM

#### Well diffusion method

The bacterial cultures used in this study included American Type Culture Collection (ATCC) standard strains, as well as reference strains of the Gram-positive bacteria *Staphylococcus aureus* (ATCC 25923) and *Bacillus subtilis* (ATCC 6633), and the Gram-negative bacteria *Escherichia coli* (ATCC 8739) and *Pseudomonas aeruginosa* (ATCC 9027). These strains were freshly grown in nutrient broth to evaluate the antibacterial activity of pomegranate using the agar well diffusion method. The bacterial strains were mixed with Mueller-Hinton Agar (MHA) and poured into petri dishes to prepare for the experiment. Once the agar had solidified, wells (5 mm) were made in the agar and filled with various concentrations of POM in DMSO (1000, 500, 250, 125, and 62.5 µg/mL). The plates were then incubated at 37 °C for 24 h. DMSO served as a control. After incubation, the zones of inhibition were measured and recorded in millimeters. All experiments were conducted in triplicate to ensure accuracy^38^.

#### The minimum inhibitory concentration (MIC) and the minimum bactericidal concentration (MBC)

Using the broth dilution method in a 96-well plate, we evaluated the minimum inhibitory concentration (MIC) and minimum bactericidal concentration (MBC) of POM against four bacterial strains (both Gram-positive and Gram-negative). The MIC was recorded as having the lowest concentration that inhibited bacterial growth. To determine the MBC, samples were streaked onto MHA; after 24 h of incubation, the MBC was identified as the concentration at which no visible bacterial growth occurred^38^.

### Regeneration study

One hundred milligrams of POM were mixed and irradiated in a microwave with a 20 mL Pb(II) solution (0.1 M) at pH 7 for 20 s, followed by separation. For the desorption process, 10 mL HCl (1 mol/L) was added, and shaken for 30 min, followed by separation, washed three times with distilled water, and finally dried at 30 °C. The adsorption process was conducted, and the metal capacity was calculated for five cycles. Similarly, the exact process was applied to PO. In the case of TC, 100 mg of POM was mixed with 10 mL of TC (10 ppm) at pH 4, followed by 30 min of shaking. The mixture was then separated. Desorption process takes place by the addition of 20 mL methanol: HCl (1:1), followed by separation, three times washing with distilled water, and drying at 30 °C. The adsorption process was conducted, and the percent removal was calculated for five cycles. Similarly, the exact process was applied to PO.

## Results and discussion

### Characterization

#### SEM of POM

**Fig. 1:**
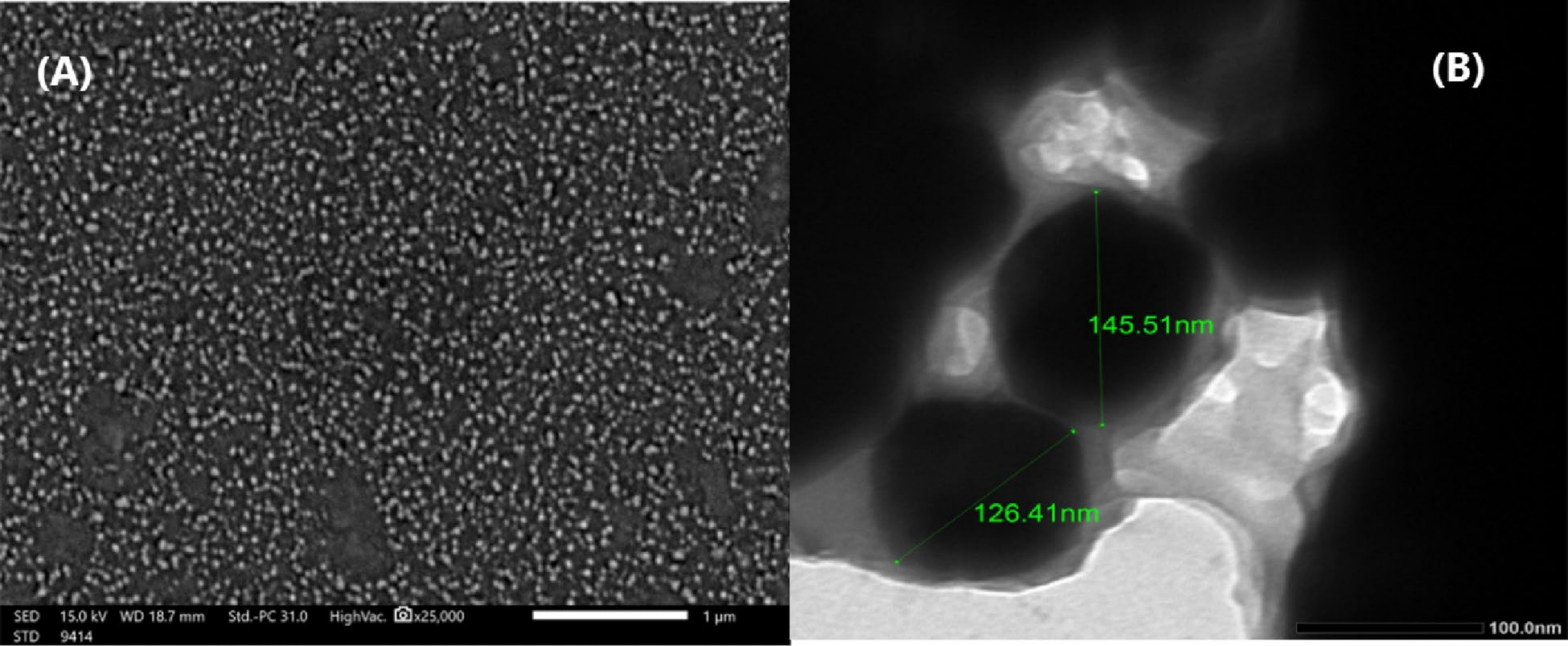
(**A**) scanning electron microscope (SEM) of POM, (**B**) Transmission electron microscope (TEM) of POM.

Scanning electron microscopy picture (Fig. 1A): The scanning electron micrograph displays a complex, hierarchical structure characterized by aggregated spherical nanoparticles. The image depicts a macroporous scaffold featuring irregular gaps that range from several micrometers to nanometer dimensions, allowing for bulk fluid infiltration during adsorption. At 25,000X magnification, additional details become discernible: the primary spherical particles exhibit a distinct spherical crystalline configuration within a continuous amorphous carbon matrix, confirming the partially ordered carbon structure. The particle surfaces exhibit considerable microscale roughness, characterized by a textured topography of fractures, ridges, and fissures that reflect the thermal degradation processes occurring during pyrolysis at 300 °C. The modification with PPE results in nanoscale topological features, characterized by densely distributed bumps or protuberances (approximately 50–100 nm) on the surfaces of the spherical units, which are attributed to the deposition of phytochemical constituents such as hydrolyzable tannins and phenolic compounds from the extract. The multi-scale surface morphology, which includes macropores, crystalline domains, amorphous regions, and nano-texturing, generates a substantial and accessible surface area along with numerous structural defects crucial for the efficient binding of Pb(II) and TC. Fast ion diffusion to active sites and strong adsorption through exposed functional groups are both made possible by this arrangement.

#### TEM of POM

Figure 1B shows a transmission electron micrograph image, confirming the nanospherical morphology observed in the scanning electron micrograph. The particles in the micrograph exhibit a very uniform size distribution, with diameters ranging from 126 to 145 nm. As a result of the applied pyrolysis conditions, the particles have solid-core architectures with no hollow interiors, indicating that the pomegranate peel precursor material has undergone complete chemical transformation. Lattice fringes inside the particles may be resolved in high-magnification regions, which demonstrates the presence of localized graphitic ordering. The interlayer spacing is measured to be around 3.14 Å between the layers. A distinct core-shell architecture is the result of the modification process. This architecture is characterized by darker, electron-dense amorphous coronas or shells that are roughly 5–10 nm thick and uniformly encapsulate the graphitic cores. It is believed that these shells were able to successfully graft phytochemical components from the PPE onto the surface of the charcoal. Additionally, the shell sections exhibit a disordered nanoporous structure with pore widths ranging from 2 to 5 nm, which falls within the mesoporous range. These properties make them effective traps for TC and Pb(II) ions.

#### X-Ray diffraction

**Fig. 2:**
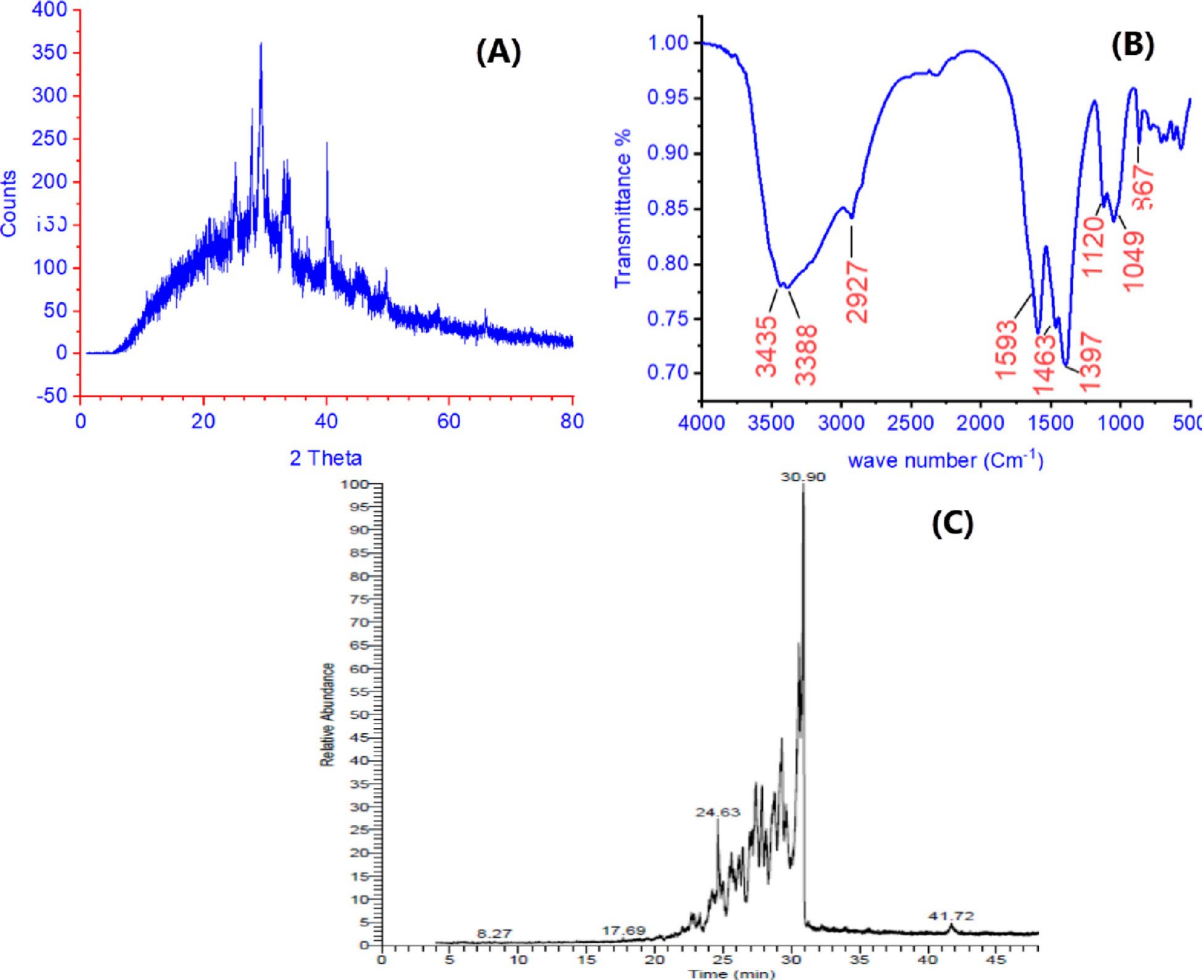
(**A**) X- Ray Diffraction (XRD) of POM, (**B**) FT-IR of POM, (**C**) GC-Ms of pomegranate extract.

The X-ray diffraction (XRD) analysis of POM reveals critical insights into its structural characteristics and adsorption-active phases. The diffraction pattern (Fig. 2A) displays a broad hump centered around 2θ = 23–28°, accompanied by weaker reflections at 42.5°, characteristic of partially graphitized carbon with a significant amorphous content^39^. The prominent peak at 28.4° corresponds to the (002) crystallographic plane of turbostratic carbon JCPDS Card 00–041-1487^40^, indicating short-range ordering of graphene-like sheets with an interlayer spacing of approximately 3.14 Å - slightly larger than perfect graphite (3.35 Å) due to heteroatom incorporation (O, N, S) from the pomegranate peel modification^41^. The secondary peak at 42.5° (100 plane) JCPDS Card 75–1621^42^ indicates that the hybridized carbon domains are structured in an in-plane manner^39^. Broad, low-intensity peaks indicate that most of the biochar is amorphous, which is beneficial for adsorption, as it suggests the presence of numerous structural flaws and edge sites that can bind TC and Pb(II). The lack of sharp crystalline peaks signifies the complete carbonization of the pomegranate peel precursor at a pyrolysis temperature of 300 °C, with no observable inorganic crystalline phases present. The transitional structure from amorphous to graphitic form establishes an optimal equilibrium between the accessibility of active sites, provided by amorphous regions, and electron conductivity, derived from graphitic domains. This configuration enhances both electrostatic interactions and weak surface complexation with TC and Pb(II) ions.

#### FT-IR spectrum

The FT-IR spectrum of POM (Fig. 2B) provides essential molecular-level evidence for its enhanced adsorption capabilities for TC and Pb(II), highlighting the functional groups introduced through modification with PPE. The band at 3435 cm⁻¹ indicates overlapping N-H stretching from aliphatic amines in peel extract alkaloids and O-H stretching from phenolic and carboxylic groups in hydrolyzable tannins, both serving as proton donors for metal coordination^39^. Peaks at 2927 cm⁻¹, corresponding to N-H deformation, and 1593 cm⁻¹, associated with asymmetric N-O stretching in nitro compounds, confirm the presence of nitrogenous ligands that facilitate covalent binding with TC and Pb(II). The prominent band at 1397 cm⁻¹ is attributed to the bending of phenolic O-H groups^43^. A high-affinity site that deprotonates at pH > 5 to generate electron-rich phenolate anions (C₆H₅O⁻), facilitating robust chelation with TC and Pb²⁺ by ionic bonding. Peaks at 1120 cm⁻¹ (C-O stretching in aliphatic ethers) and 1049 cm⁻¹ (S = O bending in sulfoxides) suggest that sulfur and oxygen heteroatoms promote soft acid-base interactions and stabilize dipole-cation interactions^43^. The presence of carbonaceous structures that enhance hydrophobicity and pore accessibility is indicated by lower-frequency bands at 1463 cm⁻¹ (C-H methylene bending), 867 cm⁻^1^ (aromatic C-H deformation), and 787 cm⁻¹ (alkene C = C vibration). The findings validate that PPE grafting enhances POM with synergistic binding sites, where amines and phenols primarily facilitate adsorption via Lewis acid-base coordination and ion exchange, whereas sulfoxides and ethers offer secondary physisorption routes. The lack of a carbonyl stretch (1700 cm⁻¹) corresponds with thermal decarboxylation occurring during pyrolysis at 300 °C, hence concentrating more stable O/N/S functionalities.

#### GC-MS analysis of PPE

The GC-MS chromatogram of PPE (Fig. 2C) displayed a complex phytochemical profile characterized by high-probability compounds at significant retention times, indicating the peel’s extensive metabolic diversity. At RT 20.57 min, the analysis identified 9,12,15-Octadecatrienoic acid, 2-[trimethylsilyloxy]−1-[[trimethylsilyloxy]methyl] ethyl ester (Probability: 7.29%, Area%: 0.08%, C₂₇H₅₂O₄Si₂, MW 496), a silylated omega-3 fatty acid derivative that indicates lipid oxidation. RT 22.02 min identified Hexadecanoic acid, 1-(1-methylethyl)−1,2-ethanediol ester (Probability: 6.24%, Area%: 0.30%, C₃₇H₇₂O₄, MW 580), a branched-chain fatty acid ester typical of cuticular waxes. The peak observed at RT 24.63 min (Probability: 10.74%, Area%: 4.27%) is attributed to Tetradecanoic acid, 12-methyl-, methyl ester (C₁₆H₃₂O₂, MW 256), a methyl-branched lipid known for its antioxidant properties. This peak co-elutes with its isomers, Cyclopentaneundecanoic acid, methyl ester (Probability: 10.32%) and Pentadecanoic acid, 14-methyl-, methyl ester (Probability: 9.52%). At RT 27.43 min, 3-Octadecanone (Probability: 15.89%, Area Percentage: 8.16%, C₁₈H₃₆O, Molecular Weight: 268) was identified as a significant long-chain ketone, presumably resulting from lipid peroxidation. The nitrogenous alkaloid-like compound 1 H-Imidazo[4,5-c] pyridine, 2-(3,4-dimethoxyphenyl), was predominant at retention time 28.75 min (Probability: 22.05%, Area Percentage: 6.22%, Molecular Formula: C₁₄H₁₃N₃O₂, Molecular Weight: 255), indicating the presence of bioactive heterocyclic constituents. Cyclohexanol, 2,4-bis(1,1-dimethylethyl)-, is the most abundant compound, detected at RT 30.90 min (Prob: 7.57%, Area%: 19.12%, C₁₄H₂₈O, MW 212), indicating the presence of sterol-like antioxidants or terpenoid degradation products. RT 32.22 min included 9-Octadecenoic acid, (2-phenyl-1,3-dioxolan-4-yl) methyl ester, cis- (Prob: 18.79%, Area%: 0.06%, C₂₈H₄₄O₄, MW 444), a phenolic-fatty acid conjugate representative of the antioxidant synergy found in pomegranate. Subsequent peaks include Arachidonoyl ethanolamide at RT 33.93 min (Probability: 9.14%, Area Percentage: 0.12%, C₂₂H₃₇NO₂, Molecular Weight: 347), an endocannabinoid, and Bismuthine, trimethyl- at RT 49.35 min (Probability: 12.80%, Area Percentage: 0.18%, C₃H₉Bi, Molecular Weight: 254), which indicate trace but significant metalloorganics.

The lack of signature ellagitannins indicates that acetone extraction favors the retrieval of non-polar to mid-low polarity compounds rather than polar phenolics. High-probability compounds, specifically fatty acid esters (RT 24.63 min), ketones (RT 27.43 min), and alkaloid-like structures (RT 28.75 min), correspond with the established antimicrobial and antioxidant properties of pomegranate peel, highlighting its nutraceutical potential, notwithstanding the methodological limitations in detecting polar compounds.

### SPE of POM and PO

#### Effect of pH

**Fig. 3:**
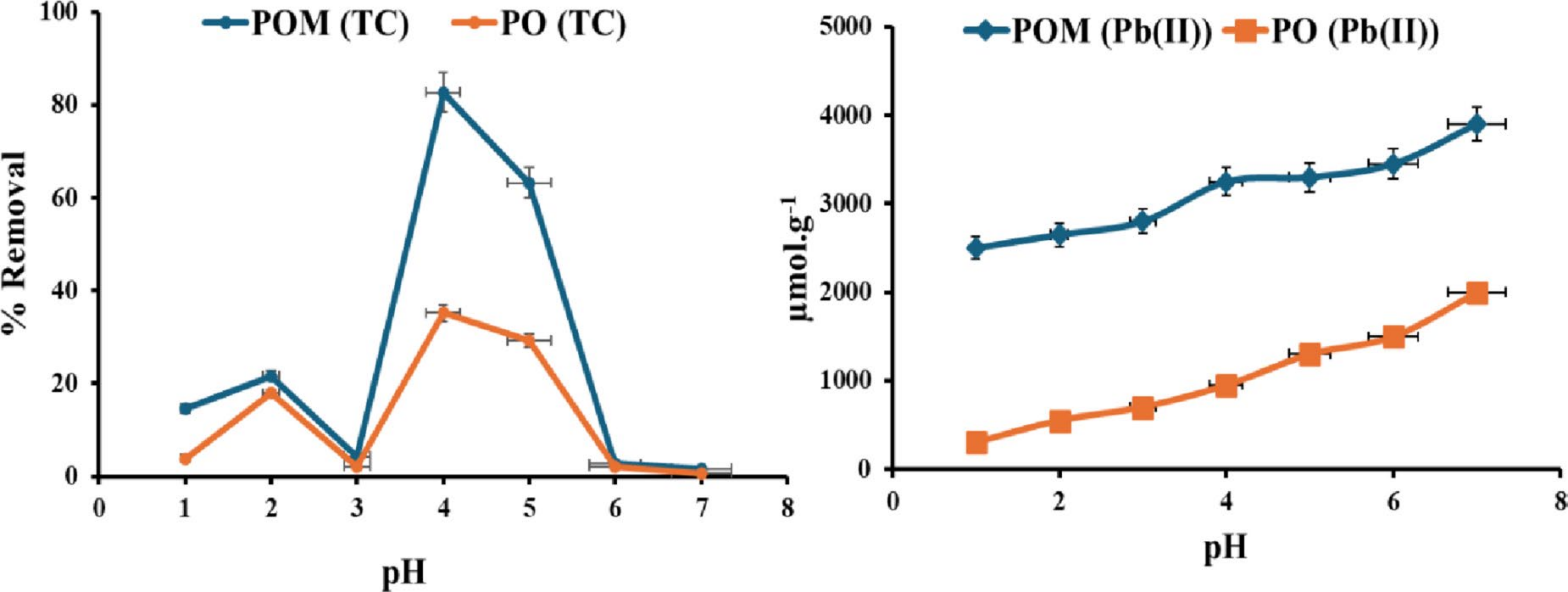
Effect of pH on adsorption of Pb(II) and TC using POM and PO.

Figure 3 illustrates the various capabilities of lead at different pH levels. Pb(II) sorption onto POM showed an increased capacity in the following order: 2500, 2650, 2800, 3250, 3300, 3450, and 3900 ± 71 (RSD 1.8%, *n* = 3) µmol/g upon pH rises. On the other hand, PO showed an increased capacity value of 300, 550, 700, 950, 1300, 1500, and 2000 ± 71 µmol/g (RSD 2.8%, *n* = 3). The maximum metal capacity value at a pH of 7 for the lead sorption trend can be defined by the formation of a chemical bond between the POM surface and the lead ions. Speciation of lead(II) in water is particularly pH-dependent. The free divalent cation, Pb²⁺, is the most prevalent species in acidic conditions (low pH). The production of mononuclear species like Pb(OH)⁺, Pb(OH)₂(aq), and Pb(OH)₃⁻, as well as polynuclear complexes like Pb₂(OH)³⁺ and Pb₄(OH)₄⁴⁺, is accompanied by hydrolysis when the pH rises^44^. Adsorption was minimal at low pH in this study. Two primary explanations exist for this phenomenon: the POM surface exhibits a high concentration of H⁺ ions that compete with Pb²⁺ for binding sites, and the positively charged adsorbent surface electrostatically repels stable, cationic Pb²⁺ molecules. At a pH of 7, acidic functional groups such as phenolic and carboxylic groups dissociate, resulting in a more deprotonated and negatively charged surface of POM. Consequently, positively charged Pb species are attracted to the surface through electrostatic forces. At pH 7, Pb²⁺ is the hydrolyzed species that exhibits the strongest attraction and retention on the negatively charged POM surface^45,46^. The pH-dependent adsorption of TC onto POM and PO for TC removal exhibits a complex relationship between the adsorbent surface chemistry, TC speciation, and electrostatic interactions. At pH 1, both adsorbents exhibit low performance, with POM reaching a removal percentage of 14.6% ± 0.18 (RSD = 0.001%, *n* = 3) and PO only 3.8% ± 1.8 (RSD = 0.03%, *n* = 3). Protonation of functional groups in acidic conditions can cause electrostatic repulsion against cationic TC species (TC⁺) or competition with abundant H⁺ ions. Increasing pH to 2 considerably boosts removal efficiency for POM (21.6% ± 0.18) (RSD = 0.001%, *n* = 3) and PO (17.8% ± 1.8) (RSD = 0.03%, *n* = 3), demonstrating a partial reduction in repulsive forces Both adsorbents show significant reduction at pH 3 (POM: 4.3% ± 0.18 (RSD = 0.001%, *n* = 3); PO: 2.2% ± 1.8 (RSD = 0.03%, *n* = 3)), perhaps because to TC’s zwitterionic transition, which hinders beneficial interactions^1^.

Adsorption efficiency reaches its peak at pH 4 for POM (82.7% ± 0.18) (RSD = 0.001%, *n* = 3) and PO (35.1% ± 1.8) (RSD = 0.03%, *n* = 3), corresponding with the prevalence of zwitterionic TC (TC⁰)^47^. This neutral structure facilitates enhanced interactions, including hydrogen bonding, van der Waals forces, and π-π stacking, while the surfaces of the adsorbents may acquire optimal charge states for binding. The improved performance of POM underlines its structural superiority, presumably related to a greater density of active sites or a stronger affinity relative to PO. At pH 5, removal diminishes although remains significant [POM: 63.2% ± 0.18) (RSD = 0.001%, *n* = 3); PO: 29.2% ± 1.8 (RSD = 0.03%, *n* = 3)], aligning with the initiation of TC deprotonation, wherein anionic species (TC⁻) arise, resulting in electrostatic repulsion from progressively unfavorable adsorbent surfaces^48^. Further pH elevation to 6–7 dramatically lowers adsorption for both materials [POM: 1.6–2.7% ± 0.18 (RSD = 0.001%, *n* = 3); PO: 0.5–2.2% ± 1.8 (RSD = 0.03%, *n* = 3)]. This trend can be characterized as follows: TC is a cation (H3L+) at low pH due to the protonation of the dimethylammonium group, but at high pH, the tri-carbonyl system and diketone fraction lose protons, converting it to anions (HL^−^ and L^2−^). Thus, these solutions are stable at neutral or mildly acidic pH levels but unstable at alkaline pH levels. At pH levels of 6–7, TC (TC⁻) and negatively charged adsorbent surfaces undergo complete deprotonation, leading to stronger repulsive forces than non-electrostatic interactions. Multifunctional binding sites in POM may explain its high adsorptive activity, as it removes TC at all pH levels. These findings suggest that pH 4 is optimal for POM-mediated TC cleanup.

#### The reaction time

**Fig. 4:**
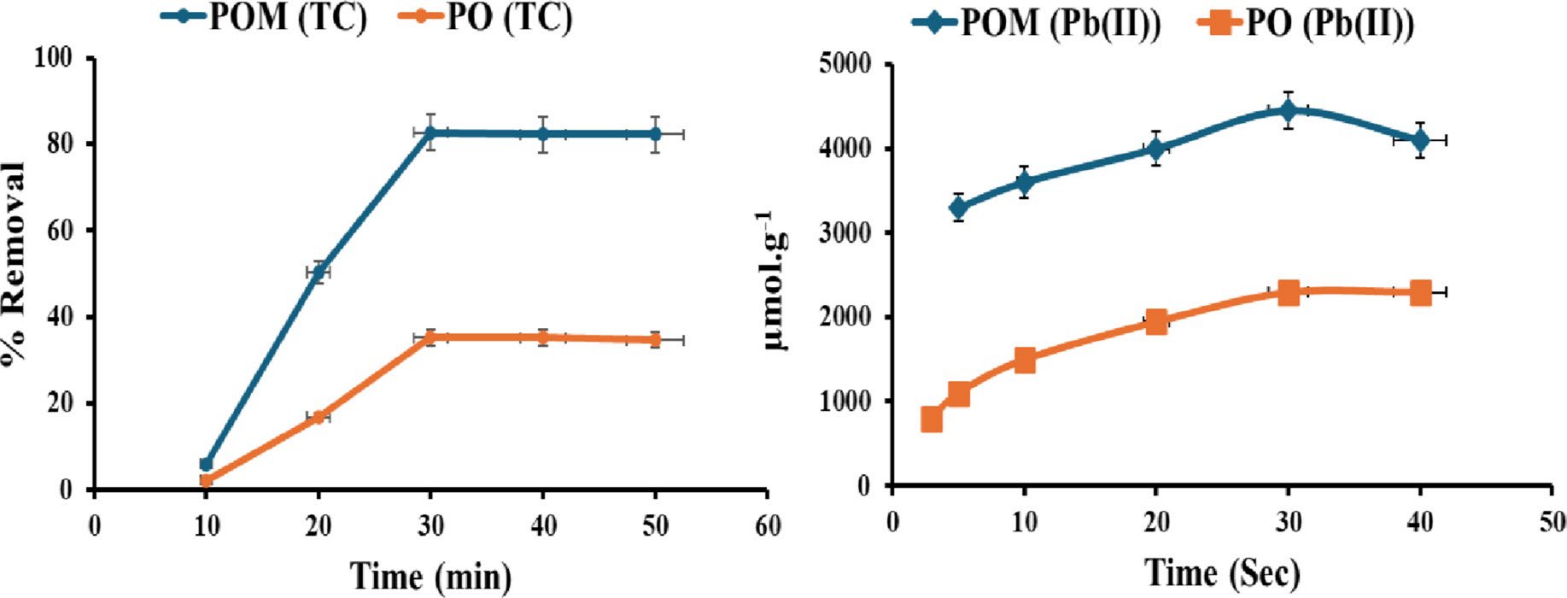
Effect of reaction time on adsorption of Pb(II) and TC using POM and PO.

For Pb(II), under microwave irradiation, the reaction time (Fig. 4) indicates a kinetically driven adsorption process characterized by rapid surface contacts and energy input restrictions. The lead adsorption capacity of the POM increased from 3250 to 4000 ± 71 µmol/g (RSD = 1.8%, *n* = 3) from 3 to 20 s, peaking at 4450 µmol/g at 30 s before decreasing to 4100 ± 71 µmol/g at 40 s due to solution boiling, a critical operational constraint. Due to microwave-specific phenomena, the molecular dipole rotation of polar functional groups (–OH, –COOH, S = O) aligns molecular dipoles with the oscillating electromagnetic field, increasing the collision frequency with Pb(II) ions. This localized thermal spotting superheats the pore surfaces of biochar, reducing diffusion resistance. Additionally, microscale cavitation collapses the vapor. Over 40 s, the PO showed a rise in capacity from 800 to 2300 ± 71 µmol/g (RSD 2.8%, *n* = 3). This ultrafast equilibrium (20s) represents the practical optimum, as more extended irradiation (30s) leads to a transient peak (4450 µmol/g) followed by a decline due to solution boiling, which is not a stable or recommended operating condition.

TC adsorption onto PO and POM exhibits unique time-dependent kinetic profiles, which are crucial for understanding their remediation methods. Both adsorbents exhibit low removal efficiency in the initial 10 min due to limited surface sites and slow diffusion of TC molecules (PO: 2.1% ± 1.8% (RSD = 0.03%, *n* = 3); POM: 5.9% ± 0.18% (RSD = 0.001%, *n* = 3)). POM has a rapid adsorption increase within 20 min (50.3% ± 0.18 (RSD = 0.001%, *n* = 3), compared to PO (16.8% ± 1.8, RSD = 0.03%, *n* = 3), indicating kinetic superiority. This acceleration may be due to increased porosity or functional groups that accelerate TC diffusion and early chemisorption. Both materials reach equilibrium within 30 min, with POM showing a higher clearance rate (82.7% ± 0.18 (RSD = 0.001%, *n* = 3)) than PO (35.1% ± 1.8 (RSD = 0.03%, *n* = 3), demonstrating significant structural changes that improve TC-binding capacity^49^.

After reaching equilibrium (40–50 min), POM exhibits near-constant adsorption (82.2% ± 0.18% (RSD = 0.001%, *n* = 3)), indicating monolayer saturation and persistent complexation. At the same time, PO displays a marginal drop (34.6% ± 1.8, RSD = 0.03%, *n* = 3), indicating weak physisorption or partial desorption. Enhanced POM performance demonstrates its ability to create high-affinity sites for irreversible TC binding via hydrogen bonding, π-π interactions, or electrostatic forces. However, POs’ plateau at lower removal indicates insufficient active sites for TC sequestration. The best POM and PO adsorption time with TC is 30 min.

**Fig. 5:**
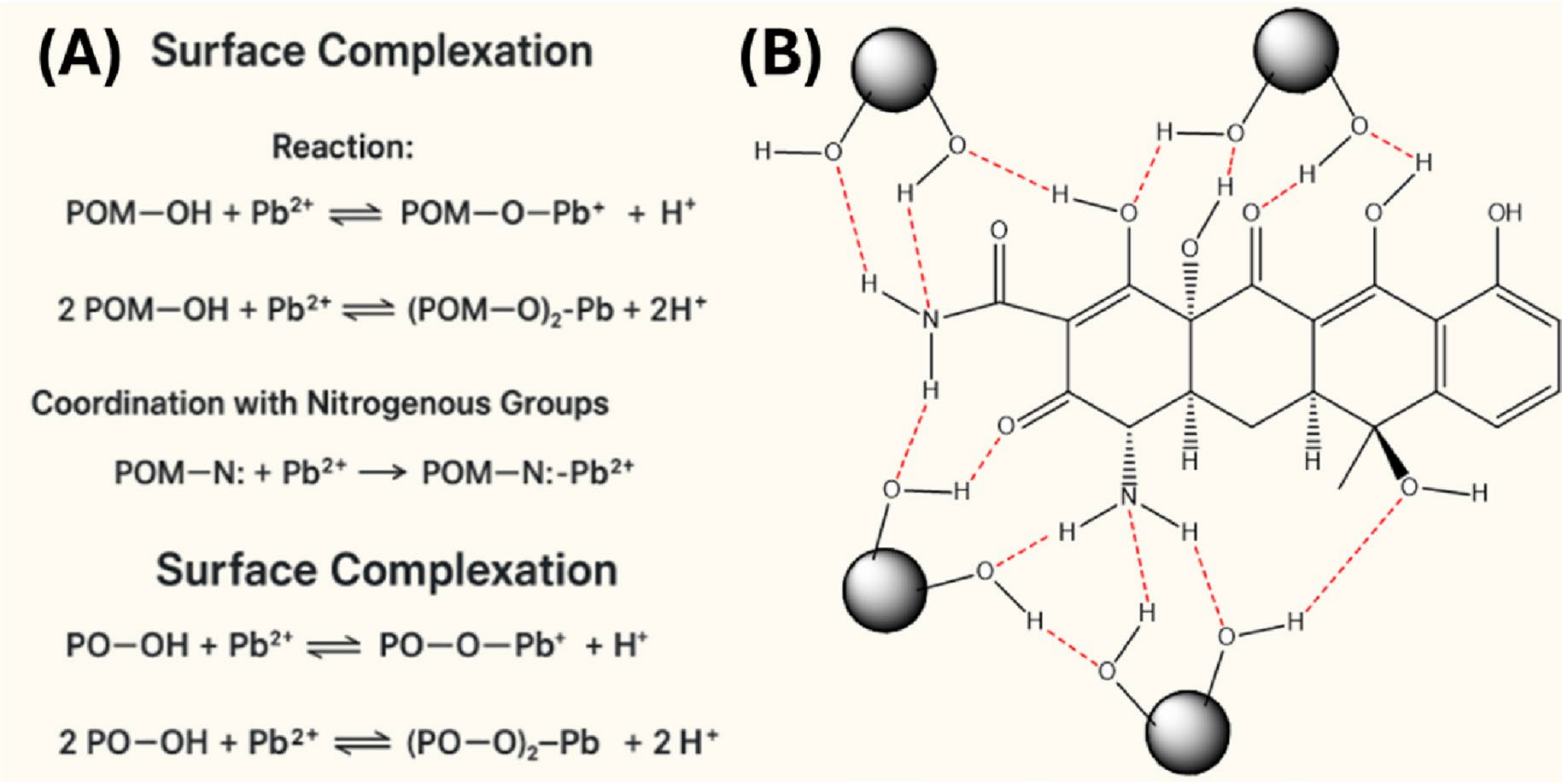
The proposed mechanisms and the corresponding reactions for the adsorption of (**A**) Pb(II). and (**B**) TC over POM and PO.

The pseudo-first-order (PFO), pseudo-second-order (PSO), Elovich, and intraparticle diffusion models were used to study Pb(II) ion adsorption onto POM and PO (Table 2). The PSO model exhibited the strongest correlation for both adsorbents, with R² values of 0.994 for POM and 0.996 for PO. This suggests that entropy-driven mechanisms, including ion exchange, electrostatic attraction, and weak surface complexation involving valence forces or electron sharing between Pb(II) ions and active surface sites, dominate the adsorption process 51]. Table 3, TC adsorption onto POM and PO kinetics. The adsorption process for both sorbents closely followed the PFO model, with perfect linearity (R² = 1.0), indicating that active adsorption sites and physisorption mechanisms predominantly determine the rate. Figure 5 describes the Pb(II) and TC adsorption processes and reactions on POM and PO. Pb(II) adsorption at pH 7 relies on surface complexation and ion exchange. The phenolic, carboxylic, and hydroxyl groups of POM and PO complex on the surface. Figure 5A shows all Pb(II)-POM/PO reactions. TC is mostly zwitterionic at pH 4, promoting hydrogen bonding. The –OH, –CONH₂, and –C = O groups of TC interact with the corresponding groups on POM and PO surfaces, as shown in Fig. 5B.

**Table 3:** Kinetic parameters of TC adsorption using POM and PO.

Pollutant	Pseudo-first-order parameters				
	**Sorbent**	**R** ^**2**^	**q**_**e**_ **(mmol/g)**	**q**_**exp**_	**k**_**1**_ **(min**^**− 1**^**)**
TC	POM	1	1.8214	0.8216	0.0871
	PO	1	0.5897	0.3486	0.0598
	**Pseudo-second-order parameters**				
	**Sorbent**	**R** ^**2**^	**q**_**e**_ **(mmol/g)**	**q**_**exp**_	**k**_**2**_ **(g/mg.min**^**− 1**^**)**
	POM	0.35	1.8214	−0.4860	0.0319
	PO	0.42	0.5897	−0.1559	0.1090
	**Elovich**				
	**Sorbent**	**R** ^**2**^	**β**	**α**	
	POM	0.9109	1.98	0.0644	
	PO	0.9086	4.48	0.0260	
	**Intra particle diffusion**				
	**Sorbent**	**R** ^**2**^	**K**_**t**_	**C**	
	POM	0.8409	0.2001	−0.4545	
	PO	0.8565	0.0895	−0.2271	

#### Dosage effect

**Fig. 6:**
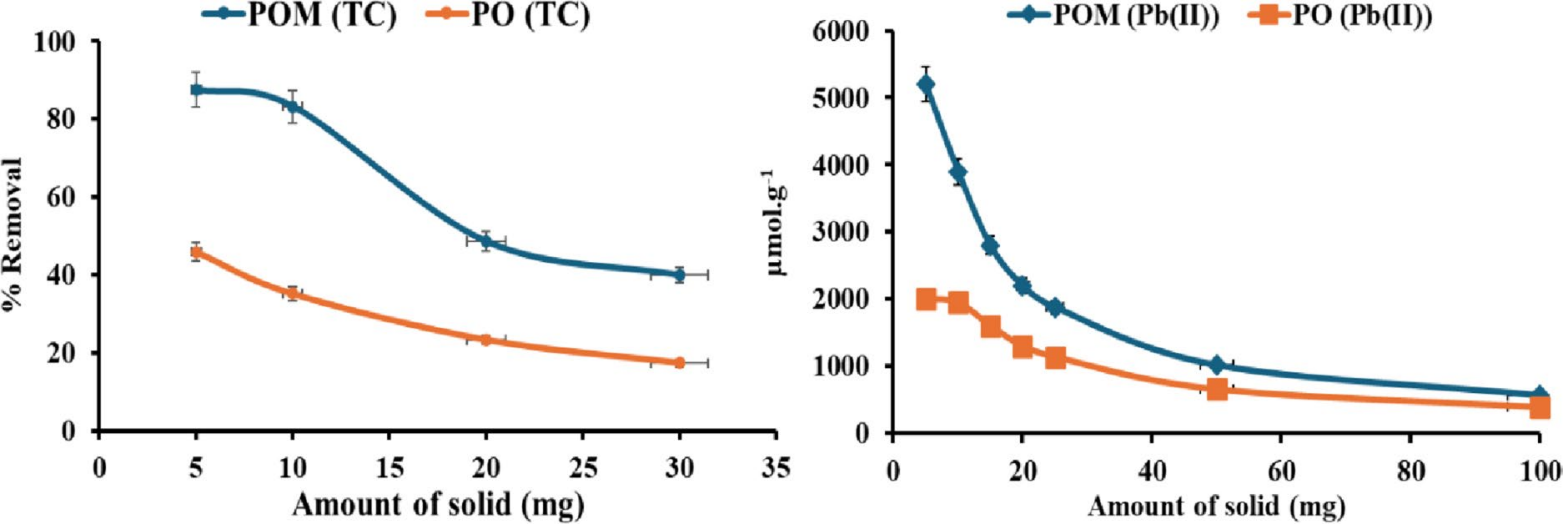
Effect of sorbent dose on adsorption of Pb(II) and TC using POM and PO.

Figure 6 illustrates the adsorption of lead onto varying amounts of POM and PO. The dose analysis reveals a fundamental inverse relationship between adsorbent mass and specific adsorption capacity, which is mediated by site accessibility dynamics and nanoscale surface heterogeneity. As the POM dosage increases from 5 mg to 100 mg, the Pb(II) adsorption capacity decreases from 5200 µmol/g to 570 ± 71 µmol/g (RSD = 1.8%, *n* = 3), a 10-fold reduction per unit mass. This occurs because fixed Pb(II) ions (0.1 M) interact with a finite pool of high-affinity sites. At ultra-low doses (5 mg), the high apparent capacity (5200 µmol/g) results from the saturation of only the highest-affinity sites by a limited Pb(II) ions. The optimal dosage of 10 mg provides the best compromise between high per-mass efficiency and practical, scalable performance, yielding a capacity of 3900 µmol/g. This preferential site utilization technique provides monolayer saturation (Langmuir behavior), resulting in peak capacity values. Critically, POM’s modification with phytochemicals enhances this Effect: its designed functional groups provide specific, high-affinity pockets that bind Pb(II) more effectively than unmodified PO (5200 vs. 2000 µmol/g at 5 mg). As the dosage increases (> 20 mg), however, site redundancy emerges, and extra biochar produces surplus adsorption sites that surpass Pb(II) availability. This dilutes ion distribution over the matrix, leaving low-energy sites unused and shifting dominance toward weaker physisorption. The diminishing performance disparity between POM and PO at high doses (570 vs. 395 µmol/g at 100 mg) suggests that modification mainly enhances binding energy rather than total site quantity. Concurrently, particle aggregation exacerbates capacity loss, since large doses encourage biochar self-association, clogging micropores and constraining diffusion paths. The ideal dosage (10 mg) thus offers a thermodynamic compromise between site scarcity-driven high efficiency with practical particle dispersion^37^.

For TC removal, the relation between adsorbent dosage and TC removal efficiency for PO and POM displays an unexpected yet mechanistically significant pattern. Unlike conventional adsorption systems, where greater adsorbent mass often promotes pollutant removal, both materials exhibit a notable negative relationship between dose (5–30 mg) and TC removal effectiveness. At the lowest dosage (5 mg), POM produces outstanding elimination [87.6% ± 0.18 (RSD = 0.001%, *n* = 3)], significantly outperforming PO [45.9% ± 1.8 (RSD = 0.03%, *n* = 3)]. This discrepancy highlights the critical importance of POM’s modification, which is likely to introduce oxygen-containing functional groups, increase the surface area, or optimize the pore structure, thereby enhancing active site availability per unit mass. As the dosage increases to 10 mg, POM retains excellent efficiency [83.2% ± 0.18 (RSD = 0.001%, *n* = 3)], whereas PO decreases abruptly [35.1% ± 1.8 (RSD = 0.03%, *n* = 3)], indicating that unaltered biochar suffers from rapid saturation or poor site usage.

The efficiency loss worsens at higher dosages (20–30 mg), with POM dropping to 48.6% and 40.0% ± 0.18 (RSD = 0.001%, *n* = 3), and PO to 23.2% and 17.3% ± 1.8 (RSD = 0.03%, *n* = 3). This behavior encourages particle aggregation and site blocking at elevated adsorbent loads. Increasing the biochar concentration increases inter-particle agglomeration, thereby reducing the accessible surface area and obstructing diffusion paths. For POM, aggregation may be exacerbated by hydrophilic functional groups introduced during modification, which encourage hydrogen bonding between particles. Additionally, greater solid concentration may generate crowding effects, where overlapping adsorption zones or reduced mixing efficiency limit mass transfer. POM’s precipitous fall relative to PO illustrates its heightened sensitivity to dosage, a trade-off for its better intrinsic activity. Notably, POM outperforms PO across all dosages, indicating that modification enhances TC affinity even under unfavorable conditions.

#### Pollutant concentration effect

**Fig. 7:**
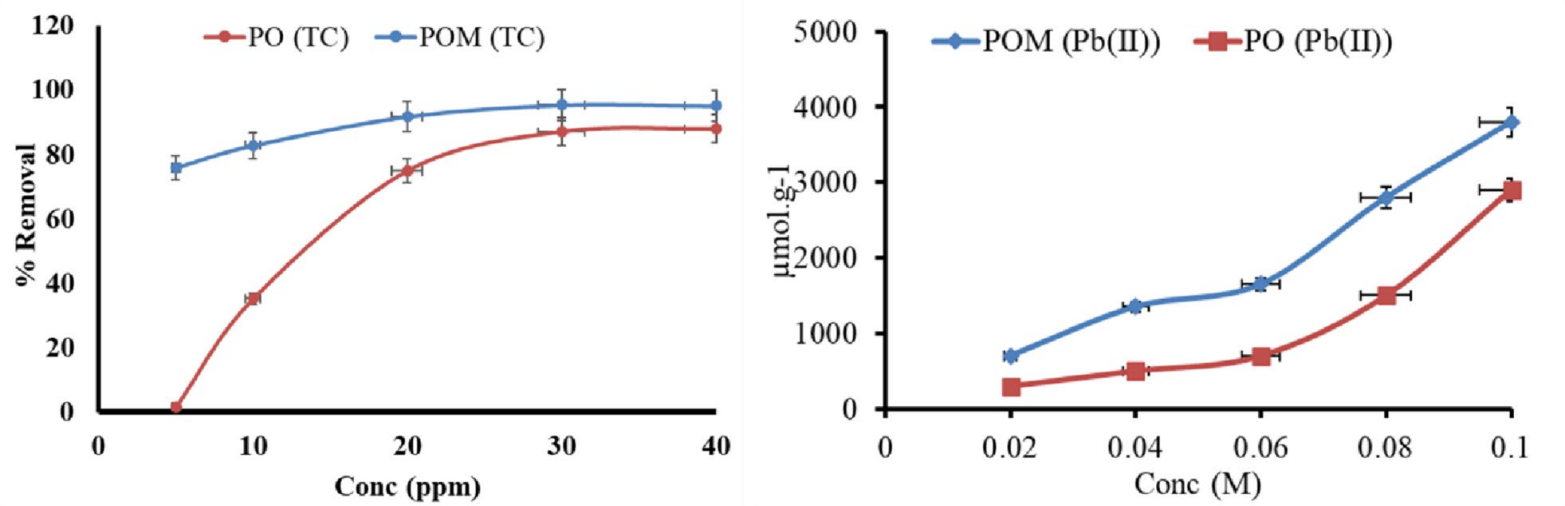
Effect of concentration on adsorption of Pb(II) and TC using POM and PO.

Figure 7 illustrates the adsorption of lead using POM and PO. The investigation into the effect of initial Pb(II) concentration on adsorption reveals a fundamental relationship governed by mass transfer dynamics, site accessibility, and the heterogeneous nature of the biochar surface. As Pb(II) concentration increases from 0.02 M to 0.1 M, the adsorption capacity of both biochar rises significantly from 700 to 3900 ± 71 µmol/g (RSD 1.8%, *n* = 3) for POM and 300 to 2500 ± 71 µmol/g (RSD 2.8%, *n* = 3) for PO. This trend is driven primarily by the enhanced concentration gradient between the bulk solution and the adsorbent surface. Pb(II) ions are insufficient at lower concentrations to saturate the abundant active sites on the biochar, resulting in underutilized capacity. The superior performance of POM across all concentrations demonstrates a higher capacity than PO directly from its modification with pomegranate peel extract, which introduces additional functional groups, as confirmed by FT-IR analysis. These groups act as high-affinity anchors for Pb(II) adsorption and complexation, particularly at lower concentrations where PO’s native sites lack sufficient binding energy.

For TC removal, the influence of initial TC concentration (5–40 ppm) on adsorption efficiency provides critical insights into the performance limits of PO and POM. TC removal efficiency increases progressively with increasing concentration for both adsorbents, though POM consistently outperforms PO across all tested ranges. At low concentrations (5 ppm), POM achieves exceptional removal [75.8% ± 0.18 (RSD 0.001%, *n* = 3)], nearly 2.3-fold higher than PO [36.2% ± 1.8 (RSD 0.03%, *n* = 3)], highlighting the efficacy of biochar modification in enhancing TC affinity through mechanisms such as surface functionalization, pore optimization, or increased active site density. This gap persists at 10 ppm [POM: 82.7% ± 0.18 (RSD 0.001%, *n* = 3); PO: 35.1% ± 1.8 (RSD 0.03%, *n* = 3)], confirming POM’s superior capacity to sequester TC under dilute conditions, likely due to the strengthened interactions imparted by modification.

As TC concentration rises to 20–40 ppm, both adsorbents exhibit increase in efficiency, but PO undergoes 74% ± 1.8 (RSD = 0.03%, *n* = 3) at 20 ppm; 87% ± 1.8 (RSD = 0.03%, *n* = 3)at 40 ppm, while POM demonstrates remarkable increase (91% ± 0.18 (RSD = 0.001%, *n* = 3) at 20 ppm; 95% ± 0.18 (RSD = 0.001%, *n* = 3) at 40 ppm. The findings reveal a significant improvement in TC adsorption efficiency as the starting concentration increases for both adsorbents, especially for the modified POM. At lower TC levels (5–10 ppm), POM eliminated roughly twice as much TC as the unmodified PO, which suggests that altering the surface of the POM made it significantly more likely to bind to TC molecules by increasing the density of active sites and optimizing the pore shape. When the TC concentration increased from 20 ppm to 40 ppm, both materials showed a clear improvement in their ability to adsorb. However, POM consistently showed a greater efficiency (91–95%) than PO (74–87%). This suggests that POM has more high-energy binding sites, improving adsorption at high solute concentrations. The synergistic effects of phytochemical functional groups, higher porosity, and enhanced π–π and hydrogen-bonding interactions between TC molecules and the modified biochar surface improve POM performance. Table 4 shows that the Langmuir model fits POM and PO best with Pb(II), indicating a monolayer adsorption mechanism. Table 5 shows that the Langmuir model fits POM and PO well. This suggests monolayer adsorption and a greater TC response.

**Table 4:** Adsorption isotherm models of Pb(II) adsorption using POM and PO.

Sorbent	Langmuir				
	**Metal ion**	**R** ^**2**^	**Q**_**0**_		**b**
POM	**Pb(II)**	0.991	0.056		42.3
PO		0.985	0.031		23.7

Sorbent	**Freundlich**				
	**Metal ion**	**R** ^**2**^		**k**_**f**_	**1/n**
POM	**Pb(II)**	0.987		0.041	0.64
PO		0.980		0.019	0.71

Sorbent	**D-R**				
	**Metal ion**	**R** ^**2**^	**ε**	**q**_**s**_	**k**_**ad**_
POM	**Pb(II)**	0.955	3.7	0.053	3.2 × 10⁻⁵
PO		0.948	3.9	0.029	3.6 × 10⁻⁵

Sorbent	**Temkin**				
	**Metal ion**	**R** ^**2**^	**A**_**t**_	**b**_**t**_	
POM	**Pb(II)**	0.972	2.94	0.010	
PO		0.963	1.81	0.008	

**Table 5:** Adsorption isotherm models of TC adsorption using POM and PO.

Sorbent	Langmuir				
	**Pollutant**	**R** ^**2**^	**Q**_**0**_		**b**
POM	**TC**	0.992	0.217		0.034
PO		0.985	0.054		0.024

Sorbent	**Freundlich**				
	**Pollutant**	**R** ^**2**^		**k**_**f**_	**1/n**
POM	**TC**	0.986		0.011	0.772
PO		0.981		0.004	0.824

Sorbent	**D-R**				
	**Pollutant**	**R** ^**2**^	**ε**	**q**_**s**_	**k**_**ad**_
POM	**TC**	0.952	4.15	0.209	2.9 × 10⁻⁵
PO		0.944	3.83	0.052	3.4 × 10⁻⁵

Sorbent	**Temkin**				
	**Pollutant**	**R** ^**2**^	**A**_**t**_	**b**_**t**_	
POM	**TC**	0.974	1.94	0.021	
PO		0.965	1.62	0.016	

#### Effect of eluent type

**Fig. 8:**
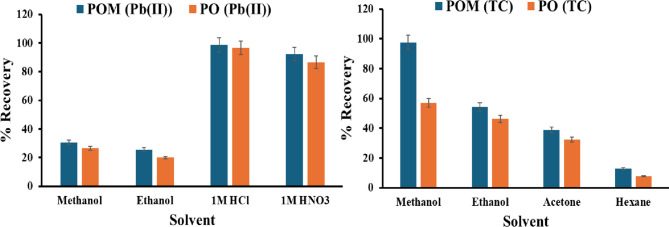
Effect of eluent type on desorption of Pb(II) and TC.

Figure 8 illustrates the desorption efficiency of Pb(II) ions from PO and POM sorbents, which was evaluated across four solvents: methanol, ethanol, 1 M HCl, and 1 M HNO₃. Both sorbents exhibited significantly higher Pb(II) recovery in acidic eluents than in organic solvents. For 1 M HCl, POM achieved near-quantitative recovery (98.7%), while PO yielded 96.7%. Similarly, 1 M HNO₃ generated 92.3% recovery for POM and 86.7% for PO. In contrast, methanol and ethanol demonstrated markedly lower efficiencies (POM: 30.8% and 25.6%; PO: 26.7% and 20.0%, respectively). This divergence underscores the critical role of anion-assisted complexation in Pb(II) desorption: HCl and HNO₃ facilitate the formation of soluble chloro- ([PbCl₄]²⁻) or nitrato-complexes, weakening Pb(II)-sorbent coordination bonds. The superior performance of POM across all solvents, particularly in acidic media, highlights enhanced ion-exchange capacity imparted by PPE modification, likely through improved Lewis acid-base interactions with Pb(II). The selection of a strong acid, such as 1 M HCl, was a strategic decision to achieve high desorption efficiency, despite the potential risk of surface damage under acidic conditions. The primary mechanisms, as previously discussed, are the protonation of active binding sites and the formation of soluble chloro-complexes, which are highly effective in disrupting the strong bonding with Pb(II). The justification for this choice is corroborated by the successful regeneration over multiple cycles, which demonstrates its practical resilience to short-term acidic exposure and confirms a viable trade-off between maximum recovery and long-term adsorbent stability.

The recovery efficiency of TC from PO and POM was systematically evaluated using five solvents: methanol, ethanol, acetone, and hexane. Experiments employed a fixed volume (5 mL) and shaking parameters (30 min, 200 rpm). Methanol achieved near-complete TC removal (97.5% and 56.9%) for POM and PO, respectively, attributable to its high polarity (dielectric constant ε = 32.7), protic nature, and strong hydrogen-bonding capacity, which disrupts TC-sorbent interactions. Ethanol (ε = 24.3) showed moderate efficiency (54.3% and 46.1%), while acetone (ε = 20.7) yielded lower extraction rates (38.8% and 32.8%), and hexane (ε = 1.9) exhibited significantly lower extraction (12.9% and 7.6%). This trend aligns with the Hildebrand solubility parameter (δ) of TC, which closely matches methanol (δ = 29.7) and ethanol (δ = 26.0). POM consistently outperformed PO across all solvents, likely due to enhanced ionic interactions between TC’s amine/carbonyl groups and the phosphomolybdate’s Lewis acid sites, facilitating selective desorption. This confirms that methanol is the most suitable solvent for the desorption process in the TC process.

#### Effect of eluent volume

**Fig. 9:**
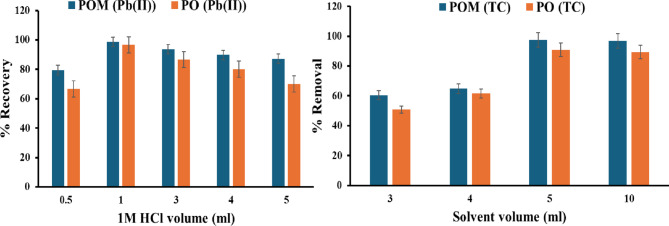
Effect of eluent volume on desorption of Pb(II) and TC.

Figure 9 shows the influence of HCl volume (0.5–5 mL of 1 M HCl) on Pb(II) recovery from PO and POM sorbents, which was evaluated under fixed shaking conditions (30 min, 200 rpm). Both sorbents exhibited a nonlinear relationship between eluent volume and desorption efficiency, with peak recovery occurring at an eluent volume of 1 mL of HCl. For POM, recovery reached near-quantitative levels (98.7%) at 1 mL but declined sharply to 93.6% (3 mL), 89.7% (4 mL), and 87.2% (5 mL). Similarly, PO achieved maximal recovery (96.7%) at 1 mL, followed by a drop to 86.7% (3 mL), 80.0% (4 mL), and 70.0% (5 mL). This volumetric threshold reflects two competing mechanisms: At low volumes (≤ 1 mL), concentrated Cl⁻ ions optimize the formation of soluble chloro-complexes, displacing Pb(II) from sorbent sites via ligand exchange. While Excess volume (> 1 mL) dilutes Cl⁻ concentration, reducing complexation efficiency and increasing the eluent-to-sorbent ratio, which promotes Pb(II) re-adsorption due to decreased ionic strength. POM’s superior efficiency retention at higher volumes (87.2% vs. PO’s 70.0% at 5 mL) underscores its structural resilience to dilution effects, attributed to phosphomolybdate’s high-density active sites maintaining affinity even under suboptimal eluent conditions.

For TC recovery, optimization of the methanol volume for the solid-phase extraction process (0–10 mL) revealed a nonlinear relationship with TC. For both sorbents, recovery increased until 5 mL, then plateaued at 10 mL, with POM reaching 60.4–97.5% and PO achieving 50.7–90.7%. This saturation suggests complete desorption of TC molecules and exhaustion of accessible sorption sites. The initial increase (1–5 mL) reflects enhanced mass transfer resulting from improved solvent penetration into the sorbent pores. Furthermore, the optimum volume of methanol required for the desorption process of TC using POM and PO is 5 ml.

#### Effect of desorption time

**Fig. 10:**
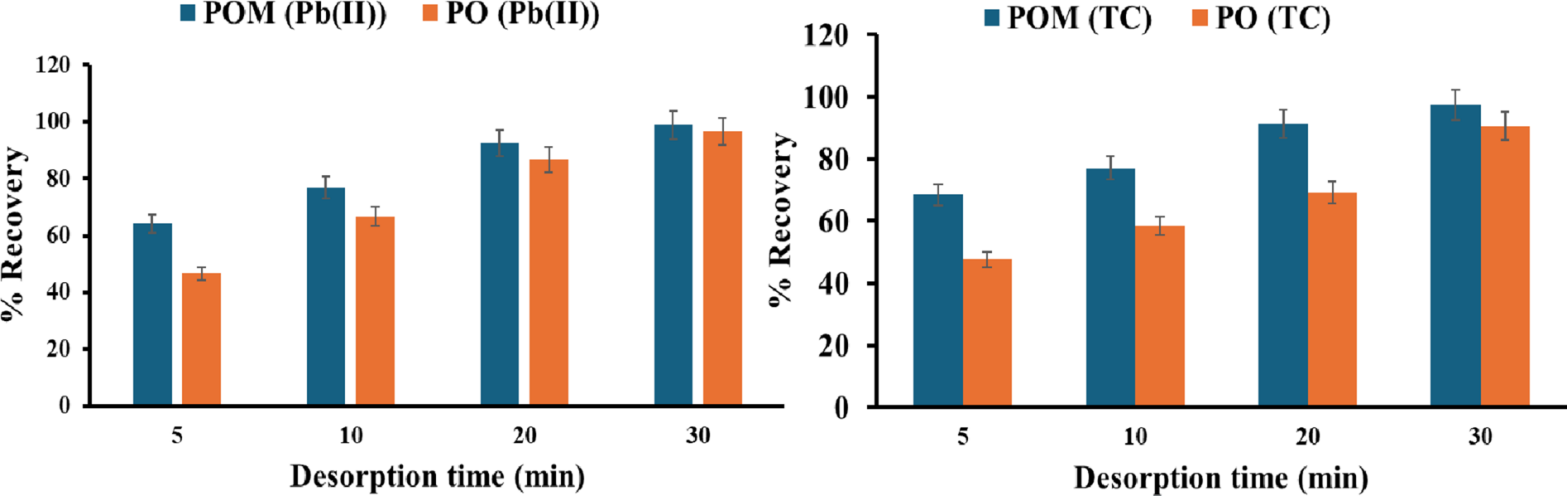
Effect of desorption time of Pb(II) and TC.

In Fig. 10, the kinetics of Pb(II) desorption from PO and POM sorbents were investigated using 1 M HCl across shaking durations of 5–30 min. Both sorbents exhibited time-dependent recovery, though POM demonstrated significantly accelerated kinetics. At 5 min, POM achieved 64.1% recovery, surpassing PO (46.7%) by 17.4%. This gap narrowed at 10 min (POM: 76.9%; PO: 66.7%) and 20 min (POM: 92.3%; PO: 86.7%), converging near saturation at 30 min (POM: 98.7%; PO: 96.7%). The rapid early-stage POM desorption (5–20 min) aligns with fast boundary-layer diffusion, where Cl^−^ ligands swiftly displace surface-bound Pb(II) ions. In contrast, PO’s slower kinetics suggest intraparticle diffusion limitations, requiring prolonged agitation to access Pb(II) bound in deeper sorbent matrices. The near-quantitative recovery for both sorbents at 30 min confirms the thermodynamic favorability of HCl-mediated desorption, while POM’s kinetic superiority (92.3% recovery at 20 min vs. PO’s 86.7%) underscores its enhanced mass transfer efficiency.

For TC desorption studies (5–30 min shaking, 200 rpm), rapid equilibration was observed, with more than 90% of the maximum recovery occurring within 30 min. Near-complete desorption (97.5% and 90.7%) was achieved using POM and PO, respectively, at 30 min. This indicates a diffusion-limited process, where initial fast desorption corresponds to surface-bound TC and slower release from intraparticle sites. POM’s superior kinetics underscore its faster mass transfer, likely due to reduced activation energy barriers from modified surface chemistry. Additionally, it confirms that the maximum recovery is achieved after 30 min of shaking.

#### Effect of HCl concentration

**Fig. 11:**
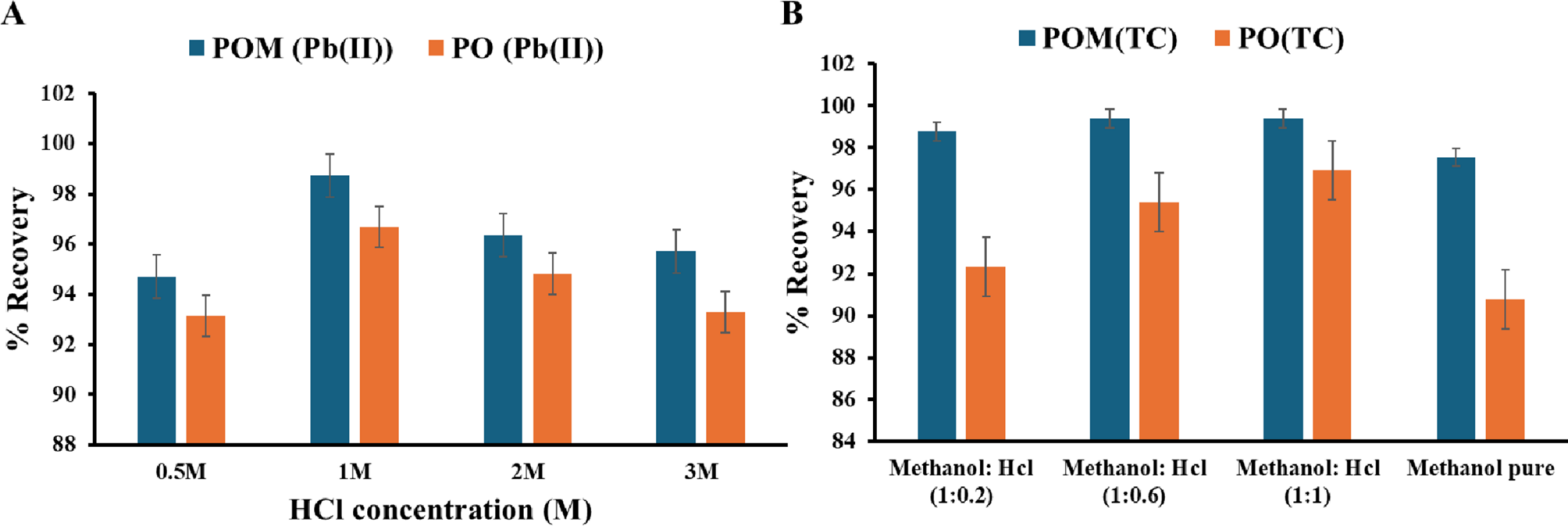
(**A**) Effect of HCl concentration on SPE of Pb(II) Recovery, (**B**) Effect of eluent acidification.

The SPE efficiency of Pb(II) using POM and PO sorbents was evaluated across HCl concentrations (0.5–3 M) under fixed conditions (5 mL eluent, 30 min shaking, 200 rpm). Both sorbents exhibited maximal recovery at 1 M HCl, with POM achieving near-quantitative desorption (98.7%) and PO reaching 96.7%. Efficiency declined marginally at higher concentrations: for POM, recovery decreased to 96.3% (2 M) and 95.7% (3 M), while PO declined to 94.8% (2 M) and 93.3% (3 M). Similarly, at a suboptimal concentration of 0.5 M HCl, POM (94.7%) outperformed PO (93.1%) as shown in Fig. 11A. This concentration-dependent trend is attributed to two competing mechanisms: Optimal chlorocomplex Formation. At 1 M HCl, Cl⁻ ions saturate Pb(II) coordination sites, forming soluble [PbCl₄]²⁻ complexes that efficiently displace Pb(II) from sorbent binding sites via ligand exchange. Additionally, competitive protonation at high [H⁺] Concentrations (> 1 M) introduces excess H⁺, which competes with Pb(II) for anionic binding sites, thereby reducing ion-exchange efficiency^50^.

#### Effect of eluent acidification

Acidification of methanol with HCl significantly enhanced TC desorption by altering solute protonation states (Fig. 11B). Pure methanol recovered 97.5–90.7% using POM and PO, respectively, while methanol: HCl mixtures (1:0.2, 1:0.6, 1:1 v/v) improved efficiency to 92–102%. The optimal 1:1 ratio yielded peak performance (98.7–99.3% for POM), (92.3–96.9% for PO) as shown in Fig. 11B, attributable to two synergistic effects: Protonation of TC: At low pH (HCl-induced), TC’s tertiary amine group becomes protonated, increasing solubility and weakening electrostatic/hydrogen-bonding interactions with sorbent surfaces. Ion-pair disruption: HCl anions compete with TC for binding sites on POM centers, accelerating desorption via ion exchange. This result confirms the positive effect of HCl on the recovery process of TC, and the optimum ratio to achieve higher recovery is a methanol: HCl mixture (1:1).

#### Analytical performance

The limits of detection (LOD) and quantification (LOQ) were determined using the standard error of the calibration curve, a standard statistical approach for estimating method sensitivity. The calculation was based on a calibration curve constructed from eleven data points (*n* = 11), each point measured in triplicate (3 times), which provided a slope of 0.0424 and a standard deviation of the intercept of 0.0222, as shown in Table 6. The resulting LOD of 1.72 and LOQ of 5.23, respectively, correspond to confidence levels of 95% for LOD and ensure a precision suitable for quantification of LOQ. This confirms the calibration curve’s high degree of confidence and the method’s suitability for detecting and quantifying low analyte concentrations. Moreover, in the case of Pb(II) determination, the objective in this work was to stress-test the adsorbents (POM and PO) and determine their saturation capacities. This requires working with high initial concentrations of Pb(II) (0.02–0.1 M) to occupy all active sites on the adsorbent fully. The titration method, with its excellent linearity (R² = 0.9959) in this high concentration range, is ideally suited for accurately quantifying these concentrations. The sensitivity of the method was evaluated through the statistical determination of the limit of detection (LOD) and limit of quantification (LOQ) using the standard error of the calibration curve. The calibration curve for Pb(II) was constructed using six concentration points, each measured in triplicate (*n* = 3), resulting in a highly linear relationship (R² = 0.9959) with a slope of 64.049. Based on the standard deviation of the y-intercept, the LOD and LOQ were calculated to be 16.27 ppm and 50.06 ppm, respectively, as shown in Table 7. These values, derived from a robust dataset where each calibration point was repeated three times, confirm the method’s excellent sensitivity and reliability for the trace-level determination of Pb(II). The reported LOD and LOQ statistically validate the precision and reliability of the method within its intended operational range for capacity calculation.

**Table 6:** Statistical data for calculation LOD and LOQ for TC.

Concentration of TC (ppm)	0.05	0.1	0.5	1	2	3	5	10	20	30	50
**Absorbance**	0.004	0.006	0.02	0.029	0.055	0.087	0.169	0.388	0.826	1.269	2.1
**Standard error of intercept**			0.00668			**Standard deviation of intercept**			0.02216		
**n**	11	**R** ^**2**^	0.999	**Slope**	0.042	**LOD**	1.7247 ppm	**LOQ**		5.2265 ppm	

**Table 7:** Statistical data for calculation LOD and LOQ for Pb(II).

Concentration of Pb(II) (M)	0.02	0.04	0.06	0.08	0.1	0.2			
**Absorbance**	1.3	2.7	4.35	5.2	6.1	13			
**Standard error of intercept**	0.20934		**Standard deviation of intercept**		0.51279				
**n**	6	**R** ^**2**^	0.9959	**Slope**	64.049	**LOD**	16.27 ppm	**LOQ**	50.06 ppm

#### Effect of temperature

Table 8 shows the thermodynamic parameters for the adsorption of Pb(II) onto both POM and PO, confirming a spontaneous and endothermic process driven by an increase in entropy. The positive values of ΔH° (POM: +2.54 kJ/mol; PO: +1.04 kJ/mol) indicate that the adsorption is endothermic, which is a characteristic feature of entropy-driven adsorption dominated by ion exchange, electrostatic interactions, and weak surface complexation, with potential contributions from specific Pb-surface interactions, rather than chemisorption alone. The positive ΔS° values (POM: +50.62 J/mol·K; PO: +43.99 J/mol·K) suggest a significant increase in randomness at the solid-solution interface, likely due to the release of solvated water molecules upon the coordination of Pb(II) ions to the adsorbent surface. The negative ΔG° values at all studied temperatures, which became increasingly hostile with rising temperature, affirm the spontaneity and enhanced feasibility of the adsorption process at higher temperatures. The more favorable ΔG° values for POM compared to PO across the temperature range underscore the enhanced affinity for Pb(II) that PPE imparts. Although the pseudo-second-order (PSO) model accurately describes the kinetic data for Pb(II) adsorption on POM and PO, this alone does not prove a chemisorption mechanism. As a result, the whole process is better described as an entropy-driven adsorption dominated by ion exchange, electrostatic interactions, and weak surface complexation, with potential contributions from specific Pb-surface interactions, rather than chemisorption alone. In general, the thermodynamic parameters, especially ΔH°, proved that the primary mechanism is physisorption; nevertheless, the excellent fit to the PSO model shows that the adsorption process may incorporate specific characteristics of chemisorption or that the rate-limiting step involves sharing or exchange of electrons^51^.

**Table 8:** Thermodynamic parameters of Pb(II) and TC adsorption using POM and PO.

Pollutant	Adsorbent	T °K	ΔH∘ (kJ·mol⁻¹)	ΔS∘ (J·mol⁻¹·K⁻¹)	ΔG∘kJ/mol
Pb	**POM**	293	2.544	50.615	−12328.33
		298			−12578.60
		303			−12830.87
		308			−13042.60
		313			−13254.33
	**PO**	293	1.035	43.989	−11868.15
		298			−12103.74
		303			−12306.82
		308			−12509.91
		313			−12712.99
TC	**POM**	288	8.514	41.081	−3319.39
		293			−3460.16
		298			−3784.18
		303			−3941.71
		308			−4205.27
		313			−4273.54
	**PO**	288	1.7912513	47.7614358	−11933.87
		293			−12214.27
		298			−12460.78
		303			−12709.16
		308			−12918.89
		313			−13128.61

Additionally, Table 8 presents the thermodynamic parameters of TC onto POM, and PO was also found to be spontaneous, as indicated by the negative ΔG° values at all temperatures. However, the underlying driving forces differed. For POM, the process was endothermic (ΔH° = +8.51 kJ/mol) and associated with a positive entropy change (ΔS° = +41.08 J/mol·K). This profile suggests that TC adsorption on POM is an entropy-driven process, where the release of ordered water molecules from the hydrophobic regions of both the TC molecule and the biochar surface provides the primary driving force, overcoming the endothermic enthalpy change. In contrast, the adsorption of TC onto PO, while also entropy-driven (ΔS° = +47.76 J/mol·K), required a much smaller energy input (ΔH° = +1.79 kJ/mol). The increasingly hostile ΔG° with temperature for both adsorbents confirms that higher temperatures favor the adsorption process. The thermodynamic data for TC are consistent with the physisorption mechanisms indicated by the pseudo-first-order kinetic model, with POM’s higher ΔH° reflecting its stronger, more specific interactions with TC compared to PO.

### Antibacterial activity of POM

**Fig. 12:**
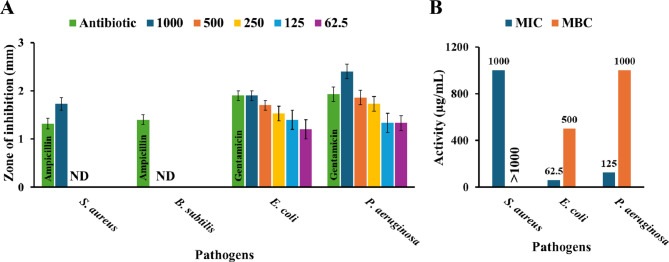
Antimicrobial activity of POM. (**A**) The inhibition zone at different POM concentrations (1000, 500, 250, 125, and 62.5 µg/mL) (mm ± SD _(*n*=3)_). (**B**) MIC and MBC. (**ND** = No detected activity).

The different POM concentrations (1000, 500, 250, 125, and 62.5 µg/mL) antibacterial activity against the 4 tested bacterial strains (*S. aureus*,* B. subtilis*,* E. coli and P. aeruginosa*) are shown in Fig. 12. In the case of the tested Gram-positive bacteria, *S. aureus* showed a small zone of inhibition (1.73 ± 0.13 mm) only at the highest concentration (1000 µg/mL), with no inhibition observed at lower concentrations, while *B. subtilis* showed no inhibition at any concentration. On the other hand, Gram-negative bacteria such as *E. coli* exhibited inhibition across all concentrations, with the largest zone (1.9 ± 0.1 mm) at 1000 µg/mL, decreasing gradually to 1.2 ± 0.2 mm at 62.5 µg/mL, while *P. aeruginosa* had the largest zones overall, with 2.4 ± 0.15 mm at 1000 µg/mL, decreasing to 1.33 ± 0.2 mm at 125 and 62.5 µg/mL. The antibiotic controls confirmed strain susceptibility, with ampicillin inhibiting Gram-positive strains and gentamicin inhibiting Gram-negative ones. Comparatively, POM showed negligible activity against Gram-positive *B. subtilis* but was marginally effective against *S. aureus* at a high concentration (1000 µg/mL). Most notably, the POM exhibited compelling activity against the Gram-negative pathogens. At 1000 µg/mL, the POM’s zone against *E. coli* (1.9 ± 0.1 mm) was identical to that of gentamicin, and its zone against *P. aeruginosa* (2.4 ± 0.15 mm) was significantly larger. This marked Gram-negative selectivity, especially against the resilient *P. aeruginosa*, suggests a promising and distinct antibacterial profile worthy of further investigation. The results of the agar well diffusion assay revealed a distinct structure-activity relationship for POM, demonstrating selective antibacterial efficacy primarily against Gram-negative pathogens. While the absolute inhibition zone sizes were modest, the direct comparison with standard antibiotics within the assay confirms a significant antibacterial effect. The POM material was largely ineffective against the Gram-positive strains, exhibiting no activity against *B. subtilis* (MIC/MBC > 1000 µg/mL) and only marginal, non-bactericidal inhibition against *S. aureus* at the highest concentration tested (MIC = 1000 µg/mL, MBC > 1000 µg/mL). In stark contrast, a concentration-dependent response was observed against the Gram-negative bacteria. For *E. coli*, POM was highly effective, with a predicted MIC of 62.5 µg/mL and an MBC of 500 µg/mL. The most significant activity was observed against *P. aeruginosa*, which exhibited the largest zones of inhibition, corresponding to a predicted MIC of 125 µg/mL and an MBC of 1000 µg/mL. This marked Gram-negative selectivity suggests that the POM’s mechanism of action may be dependent on fundamental differences in cell wall structure, such as the outer membrane composition, and positions it as a promising candidate for further investigation against resilient Gram-negative pathogens.

According to studies conducted in the past few decades, extracts from pomegranates have been the subject of multiple investigations into their potential antibacterial effects. Pomegranate peel contains polyphenols with antibacterial properties, which is why it exhibits antibacterial activity^52^. Punicalagin, gallic acid, ellagic acid, chlorogenic acid, caffeic acid, apigenin, quercetin, pelargonidin, cyanidin, granatin A, and granatin B are among the primary phenolic chemicals found in pomegranate peel, as previously described^53^.

Our observed inhibition zones at different concentrations differ from the findings of Kanatt et al., who reported that pomegranate extracts exhibited no significant activity against Gram-negative bacteria, including *E. coli*^54^. According to multiple investigations, the peel extracts have demonstrated potent, broad-spectrum efficacy against a variety of harmful microorganisms, including *Listeria monocytogenes*, *Listeria innocua*, *Staphylococcus aureus*,* Staphylococcus epidermidis*,* Pseudomonas aeruginosa*,* E. coli*,* Salmonella* sp., and *Propionibacterium acnes*^55^. Distinct variations in the antibacterial activity of the peel extracts may be attributed to the underlying chemical composition of fruit peel and intergenetic cultivar variability. As plants are malleable in their responses to geo-environmental spatial deviations, gene-to-biosynthetic changes are well established^52^. In general, the polyphenols included in pomegranates have excellent pharmacological benefits. A wide range of medicinal effects, including antibacterial, anticoagulant, liver-protective, antigenotoxic, and anti-inflammatory actions, have been discovered in these substances. The fact that these natural compounds can have a broad range of positive benefits demonstrates their power^56^.

The antibacterial efficacy of POM is attributed to the synergistic interaction of phytochemicals derived from pomegranate peel extract, primarily polyphenols and tannins, which adhere to the charcoal surface. These chemicals compromise bacterial membranes by inducing oxidative stress and interacting with cell wall proteins, resulting in the leakage of cellular contents. Their hydroxyl and carboxyl groups chelate metal ions and form hydrogen bonds with microbial enzymes, leading to enzyme deactivation and suppression of DNA replication. The pronounced efficacy against Gram-negative bacteria (*E. coli*,* P. aeruginosa*) is attributed to the facilitated penetration of small phenolic compounds through the outer membrane and their interaction with lipid constituents. In contrast, the dense peptidoglycan layer of Gram-positive bacteria restricts the diffusion of these bacteria. This method aligns with prior results indicating that pomegranate-derived polyphenols exhibit membrane-damaging and protein-denaturing effects^57^.

A significant challenge in treating wastewater co-contaminated with heavy metals and antibiotics, such as TC, is the concomitant risk of fostering ARBs^58^. Conventional sorbents, while effective for sequestrating specific pollutants, often provide a passive platform that can inadvertently enrich ARBs. These bacteria can not only proliferate but also potentially degrade the adsorbed antibiotics, thereby compromising the remediation efficiency and exacerbating the spread of antimicrobial resistance (AMR)^59^. Therefore, the development of a dual-functional sorbent that can simultaneously recover target pollutants and mitigate biological risks represents a critical advancement in environmental remediation technology. The innovation of our work lies in the design of POM, which not only facilitates the efficient recovery of Pb(II) and tetracycline but also exerts concurrent antimicrobial action. This work thus establishes a paradigm for next-generation sorbents that address both chemical and biological facets of complex pollution.

### Regeneration study

**Fig. 13:**
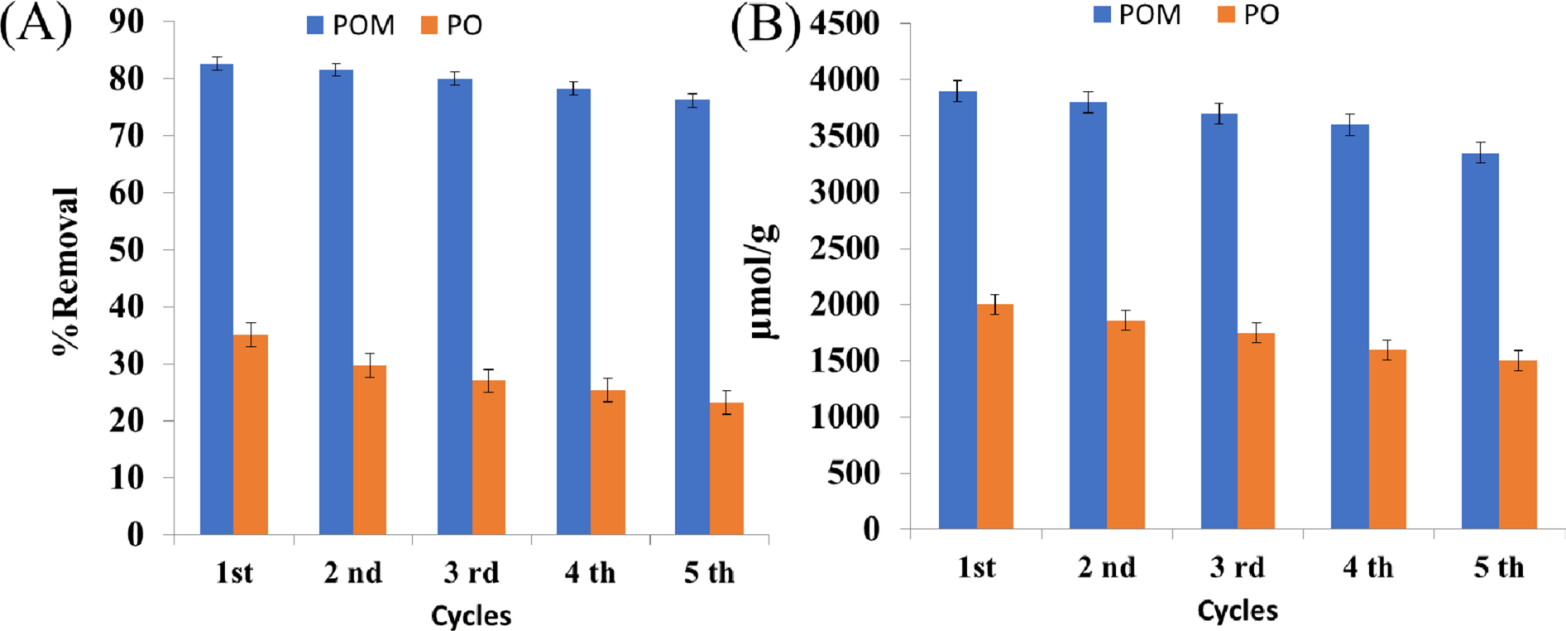
Regeneration study of POM and PO with (**A**) TC, and (**B**) Pb(II).

The percent removal was 82.70%, 81.62%, 80.00%, 78.37%, and 76.21% for POM. In comparison, the regeneration study of PO with TC was repeated for five cycles. The obtained percent removals were 35.13, 29.72, 27.03, 25.41, and 23.24%, indicating the higher efficiency of POM for the removal of Pb(II) and TC over five cycles (Fig. 13A). The regeneration study of POM with Pb(II) was repeated for five cycles (Fig. 13B). The metal capacity was 3900, 3800, 3700, 3600, and 3350 µmol/g. In comparison, the regeneration study of PO gives metal capacity values as follows: 2000, 1860, 1750, 1600, and 1500 µmol/g. In contrast, the regeneration study of POM with TC was repeated for five cycles. The gradual decrease in adsorption capacity over five cycles can be attributed to the partial leaching of functional groups or minor structural changes that occur under acidic conditions during the 1 M HCl desorption step, a common phenomenon observed in regenerable biosorbents. Nevertheless, the retention of over 86% and 75% of the original capacity for Pb(II) and the retention of over 92% and 66% of the original capacity for TC confirms that the core structure of POM and PO remains sufficiently intact for multiple uses, validating the choice of 1 M HCl and methanol: HCl (1:1) as an effective and practical eluent^60^.

The multifunctional surface of POM, characterized by its hierarchical porosity, graphitic domains, and a rich array of oxygen- and nitrogen-containing functional groups, suggests a strong potential for remediating a broad spectrum of aquatic contaminants beyond Pb(II) and tetracycline, including other heavy metals (e.g., Cd²⁺, Cu²⁺), synthetic dyes, and various antibiotics. This versatility aligns with advanced adsorbents like modified chitosan magnetic nanoparticles^44,61–64^, yet our approach distinctively leverages pomegranate peel, an abundant agricultural waste, to create a sorbent with compelling cost-effectiveness, scalability, and inherent greenness. The synthesis transforms a disposal problem into a valuable resource using simple, scalable techniques, such as pyrolysis, thereby avoiding expensive reagents and drastically reducing raw material costs compared to engineered nanomaterials. The innovation of POM lies not only in its high adsorption capacity, as contextualized by recent SPE strategies^65^, but also in its integrated dual-action: the simultaneous removal of chemical pollutants and the provision of intrinsic antimicrobial properties, a feature not common in conventional adsorbents compared to different sorbents used, as shown in Table 9. This dual functionality offers a significant economic advantage over single-purpose materials that would require sequential treatment or the use of additional biocides. Furthermore, POM’s efficient regeneration over multiple cycles enhances its long-term economic viability, while its lifecycle, from a renewable waste stream to a material that prevents secondary microbial pollution, positions it as a sustainable, multifunctional solution that aligns perfectly with the principles of green chemistry and the circular economy.

**Table 9:** Comparison of different sorbents in removal of Pb(II) and TC.

Sorbent	Pollutant	Reference
POM	3900 µmol/g for Pb(II) (pH = 7, and 20 s), 95.2% (pH = 4, 30 ppm)	This work
Pomegranate-peel/rGO nanocomposite	175.4 mg/g (Pb(II))	^64^
Rice-husk biochar (Ni@TiO₂/biochar composite)	122.3 mg/g (Pb(II))	^63^
N-doped MgO-modified corncob biochar	1429 mg/g (Pb(II))	^65^
Cow-manure biochar-vitamin-C modified (CDBC-VC)	93.69% TC removal	^62^

## Conclusion

In conclusion, this study designed and characterized POM, a novel, eco-friendly biochar, for the effective SPE of hazardous lead (Pb(II)) and the antibiotic TC from aqueous solutions. POM exhibited better adsorption capabilities for both pollutants than PO, achieving maximum adsorption under optimal conditions (pH 7 for Pb(II) and pH 4 for TC, a 30 min contact time for TC, 20 s of microwave irradiation for Pb(II), and a 10 mg sorbent dosage). POM and PO adsorption of Pb(II) obey the pseudo-second-order (PSO) model accurately; however, this alone does not prove a chemisorption mechanism. As a result, the whole process is better described as an entropy-driven adsorption dominated by ion exchange, electrostatic interactions, and weak surface complexation, with potential contributions from specific Pb-surface interactions, rather than chemisorption alone. In general, the thermodynamic parameters, especially ΔH°, have proved that the primary mechanism is physisorption; nevertheless, the excellent fit to the PSO model suggests that the adsorption process may incorporate certain characteristics of chemisorption or that the rate-limiting step involves the sharing or exchange of electrons. TC follows the first-order model, demonstrating physisorption of POM and PO. In addition, the Langmuir model fits POM and PO well with Pb(II) and TC, indicating the formation of a monolayer on the adsorbent surface. SEM, TEM, FT-IR, and XRD studies revealed that the phytochemical alteration introduced numerous functional groups and generated a core-shell nanostructure with enhanced surface characteristics, leading to superior performance. Desorption efficiencies of > 98% for Pb(II) in 1 M HCl and > 97% for TC in acidified methanol (1:1 v/v HCl: methanol) suggest reusability, as shown by the regeneration study of POM with Pb(II) and TC over five cycles. The LOD and LOQ for TC were 1.725 and 5.227 ppm, while Pb(II) ions were 16.27 and 50.06 ppm. The thermodynamic characteristics for Pb(II) and TC adsorption on POM and PO show a spontaneous, endothermic process driven by entropy. Furthermore, POM exhibited notable antibacterial activity against Gram-negative bacteria, demonstrating potency comparable to that of a standard antibiotic and meriting further investigation. These results highlight POM’s dual functionality as a highly effective, sustainable, cost-effective sorbent for simultaneous heavy metal and antibiotic remediation in water treatment applications, leveraging valorized waste biomass.

## Data Availability

The authors confirm that the data supporting the findings of this study are available within the article.
